# A Computational and Chemical Design Strategy for Manipulating Glycan‐Protein Recognition

**DOI:** 10.1002/advs.202308522

**Published:** 2024-04-06

**Authors:** Qiang Zhu, Didi Geng, Jingchao Li, Jinqiu Zhang, Haofan Sun, Zhiya Fan, Jiahui He, Ninghui Hao, Yinping Tian, Liuqing Wen, Tiehai Li, Weijie Qin, Xiakun Chu, Yong Wang, Wen Yi

**Affiliations:** ^1^ Departments of Biochemistry & Biophysics College of Life Sciences Zhejiang University Hangzhou 310012 China; ^2^ National Center for Protein Sciences Beijing State Key Laboratory of Proteomics Beijing Proteome Research Center Beijing Institute of Lifeomics Beijing 100026 China; ^3^ The Provincial International Science and Technology Cooperation Base on Engineering Biology Shanghai Institute for Advanced Study Institute of Quantitative Biology International Campus of Zhejiang University Haining 314499 China; ^4^ Carbohydrate‐Based Drug Research Center Shanghai Institute of Materia Medica Chinese Academy of Sciences Shanghai 201203 China; ^5^ Advanced Materials Thrust Function Hub The Hong Kong University of Science and Technology Guangzhou 511400 China; ^6^ Cancer Centre Zhejiang University Hangzhou 310012 China

**Keywords:** CH–π interaction, glycan‐protein recognition, glycoproteomics, PhoSL, core fucose

## Abstract

Glycans are complex biomolecules that encode rich information and regulate various biological processes, such as fertilization, host‐pathogen binding, and immune recognition, through interactions with glycan‐binding proteins. A key driving force for glycan‐protein recognition is the interaction between the π electron density of aromatic amino acid side chains and polarized C─H groups of the pyranose (termed the CH–π interaction). However, the relatively weak binding affinity between glycans and proteins has hindered the application of glycan detection and imaging. Here, computational modeling and molecular dynamics simulations are employed to design a chemical strategy that enhances the CH–π interaction between glycans and proteins by genetically incorporating electron‐rich tryptophan derivatives into a lectin PhoSL, which specifically recognizes core fucosylated *N*‐linked glycans. This significantly enhances the binding affinity of PhoSL with the core fucose ligand and enables sensitive detection and imaging of core fucosylated glycans in vitro and in xenograft tumors in mice. Further, the study showed that this strategy is applicable to improve the binding affinity of GafD lectin for *N*‐acetylglucosamine‐containing glycans. The approach thus provides a general and effective way to manipulate glycan‐protein recognition for glycoscience applications.

## Graphical abstract

Computational modeling is employed to identify the critical tryptophan residue on PhoSL for recognizing core fucosylated glycans. The tryptophan residue is replaced with electron‐rich derivatives of tryptophan using the genetic code expansion strategy to enhance CH–π interactions between PhoSL and glycans. The engineered lectin is able to detect and image core‐fucosylated glycans in cells and in mice with high sensitivity.

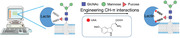

## 1 Introduction

Glycans are one of the most structurally diverse biomolecules in nature and play central roles in a range of important biological processes, including embryonic development, immune recognition, inflammatory response, and cell–cell communication.^[^ ^1^, ^2^, ^3^, ^4^ ^]^ The functions of glycans are exerted mainly through specific molecular recognition between glycans and glycan‐binding proteins (lectins). For example, the interaction between hemagglutinin proteins on influenza viruses and sialylated glycans on host cells initiates the colonization and infection process.^[^ ^5^, ^6^ ^]^ The binding of mammalian sperm to the egg is regulated mainly by the interaction between the egg‐binding protein (EBP) located on the sperm plasma membrane and the carbohydrate sequence on the egg's zona pellucida (ZP).^[^ ^7^ ^]^ Additionally, galactin‐3 regulates dendritic cell differentiation and innate immune response via interacting with cell surface β‐galactosides.^[^ ^8^, ^9^, ^10^ ^]^ Thus, glycan‐protein interactions constitute a fundamental molecular mechanism that governs glycan‐dependent biological functions.

Our current understanding of the major forces driving the glycan‐protein interactions stems from three‐dimensional structural analysis of glycan‐binding proteins and biochemical point mutational studies.^[^ ^11^, ^12^, ^13^ ^]^ These forces include bifurcated hydrogen bonds between glycan hydroxyl groups and hydrogen‐bonding amino acids, coordination of calcium‐ion with vicinal hydroxyl groups of glycans, and the interaction between electron‐rich aromatic amino acid side chains and polarized C─H groups of the pyranose (termed CH–π interactions).^[^ ^14^, ^15^, ^16^, ^17^ ^]^ Structural studies and bioinformatic analyses of glycan‐binding sites further highlight the critical role of CH–π interactions in governing glycan‐protein recognition, as aromatic amino acid residues (particularly tyrosine and tryptophan) are dramatically enriched in glycan‐binding sites.^[^ ^18^, ^19^, ^20^, ^21^, ^22^, ^23^ ^]^ This knowledge has opened up opportunities for manipulating glycan‐protein recognition to further elucidate glycan‐mediated biological processes.

Here, we employ computational modeling and molecular dynamics simulations to design a general chemical approach to increase glycan‐protein recognition by enhancing the CH–π interaction with the genetic code expansion strategy. Replacement of tryptophan residues in the glycan‐binding site with electron‐rich derivatives of tryptophan substantially increases the electrostatic potential of the indole ring, leading to enhanced CH–π interactions. We demonstrate this approach by engineering high‐affinity lectins capable of recognizing core fucosylated *N*‐linked glycans and *N*‐acetylglucosamine (GlcNAc)‐containing glycans, respectively, and further apply them for sensitive detection and imaging of glycans. This study provides a powerful tool for genetically manipulating glycan‐protein recognition and paves the way for further dissection of glycan‐dependent biological functions.

## 2 Result

### 2.1 Trp28 of Lectin PhoSL is the Most Critical Residue for Recognizing Core‐Fucosylated Glycans

**Figure 1:**
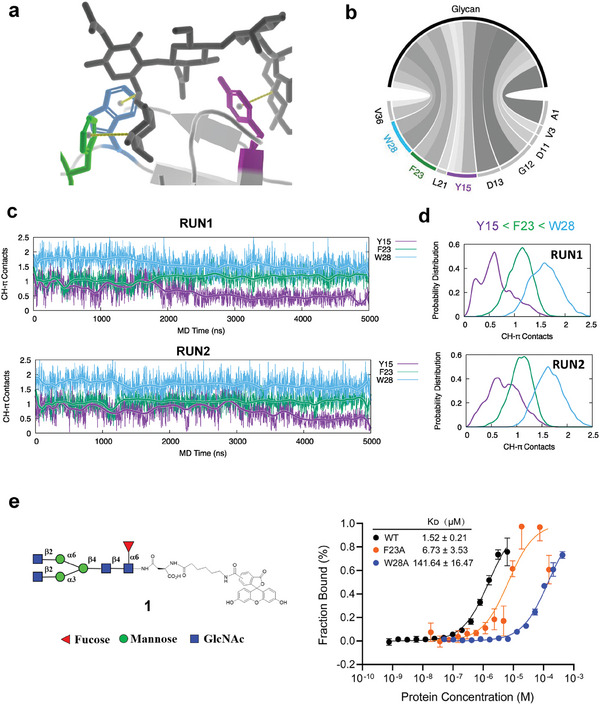
W28 is the most critical residue for glycan recognition for PhoSL. a) The energy‐minimized crystal structure of the glycan binding site of PhoSL in complex with core fucose glycan (PDB: 6FX1). It highlights the CH–π interactions formed between the aromatic residues Y15, F23, and W28 of PhoSL and the CH groups of the glycan. b) Interactions between the core fucose glycan and PhoSL residues as revealed by MD simulations. The lines connecting PhoSL residues and the glycan represent the interactions between them, and their thickness is proportional to the occurrence frequency of the corresponding interaction in MD simulations. c) The CH–π contacts between the aromatic residues Y15, F23, and W28 of PhoSL and the CH groups of the glycan, shown as a function of simulation time in two independent 5000 ns MD simulations. d) The distribution of the number of CH–π contacts formed by each residue. e) The binding affinity of the WT, F23A, and W28A PhoSL toward the core fucose glycan substrate 1 as measured by microscale thermophoresis. Error bars denote the means ± SD in three independent assays.

Core fucosylation, the addition of α1,6‐fucose to the innermost GlcNAc residue of N‐linked glycans, plays critical roles in regulating various physiological processes, including immune response, stem cell homeostasis, neuronal development, and tumorigenesis.^[^ ^24^, ^25^ ^]^ Accumulated studies demonstrated that core fucosylation is frequently upregulated in hepatocellular carcinoma, melanoma, breast and prostate cancer, and positively correlated with poor prognosis in patients.^[^ ^24^, ^26^, ^27^ ^]^ Besides, core fucosylated serum proteins also serve as promising biomarkers for disease diagnosis.^[^ ^28^ ^]^ Thus, it is important to develop strategies for the detection of core fucosylated glycans with high sensitivity. Pholiota squarrosa lectin PhoSL, isolated from a mushroom, specifically recognizes core‐fucosylated *N*‐glycans.^[^ ^29^ ^]^ Notably, PhoSL is only composed of 40 amino acids, which makes the recombinant expression and genetic manipulation of PhoSL very convenient.^[^ ^30^ ^]^ We then used PhoSL as a model system to investigate and manipulate the CH–π interaction involved in recognizing core fucosylated N‐glycans. A previously solved crystal structure of PhoSL in complex with a core fucosylated glycan ligand revealed several aromatic residues in the binding pocket, Tyr15 (Y15), Phe23 (F23), and Trp28 (W28), which interacted with core fucose and GlcNAc sugars ^[^ ^11^ ^]^ (**Figure** **1**a).

To evaluate how these residues affect glycan recognition, we built atomistic models and performed all‐atom molecular dynamics (MD) simulations in explicit solvent.^[^ ^31^ ^]^ Two 5‐microsecond simulations showed that several residues, including A1, V3, D11, G12, D13, L21, and V36, along with the aromatic residues, Y15, F23, and W28, were involved in the interactions with the glycans (Figure 1b). We also analyzed the CH–π interactions by counting the coordination number between the aromatic ring centers of these residues and the CH groups of sugars. We found stable CH–π contacts between the indole ring of W28 and the pyranose ring of GlcNAc, and between the benzene ring of F23 and the fucose ring (Figure 1c). We observed that W28 formed more CH–π contacts than F23 (Figure 1d). Moreover, Y15 exhibited weaker and less frequent CH–π contacts than F23 and W28. Thus, the simulations reveal key roles for aromatics F23 and especially W28 in CH–π–mediated glycan recognition.

To further evaluate the role of F23 and W28 experimentally, we introduced alanine mutations at these sites in PhoSL. The mutant proteins were expressed and purified from *E. coli*. We then measured their binding affinity toward the core fucose glycan substrate **1** using microscale thermophoresis (MST) (Figure S1, Supporting Information). Compared to the wildtype (WT) PhoSL, which had a dissociation constant (K_D_) of 1.52 ± 0.21 µm, the F23A mutant displayed a three‐fold weaker affinity with a K_D_ of 6.73 ± 3.53 µm. An even more substantial drop of ≈90‐fold was observed for the W28A mutant, which had a K_D_ of 141.64 ± 16.47 µm (Figure 1e). This significantly reduced binding of both mutants indicates that F23 and W28 are critical for the recognition of the glycan substrate. Moreover, the greater effect of the W28A mutant compared to F23A is consistent with MD simulations that predicted a larger contribution of CH–π contacts from W28 versus F23. Taken together, the alanine mutagenesis experiments and simulations converge to demonstrate a key role for W28 in glycan binding affinity.

### 2.2 Substitution of W28 with Electron‐Rich Tryptophan Derivatives Enhances Glycan‐Protein Recognition

CH–π interactions are important for glycan‐protein recognition, as they involve electropositive C─H bonds of glycans and the π systems of aromatic amino acid side chains. We wondered if increasing the electronic density of aromatic rings would boost CH–π interactions and the glycan binding affinity. Since W28 is more sensitive to modification than F23, we decided to choose W28 as a better candidate for manipulation. We hypothesized that the electron‐rich substitution of W28 would result in a stronger attractive force for glycan‐protein recognition.

**Figure 2:**
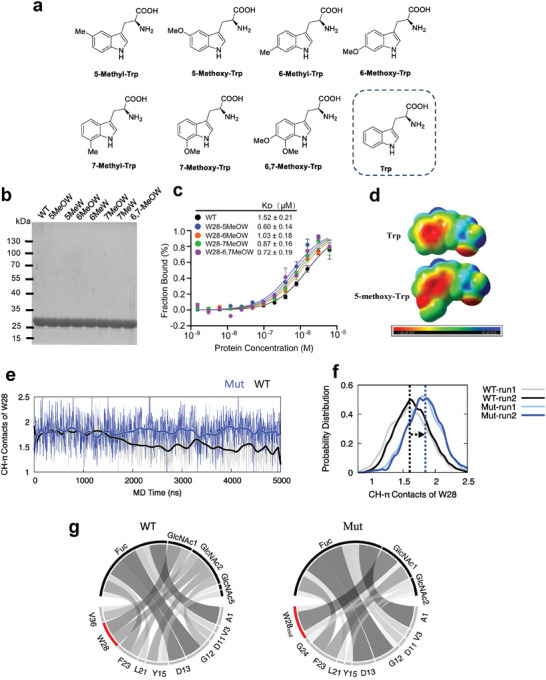
Substitution of W28 with electron‐rich tryptophan derivatives enhances glycan‐protein recognition. a) Illustrations of tryptophane derivatives for genetic incorporation into PhoSL. b) Purification of PhoSL variants containing the tryptophane derivatives. c) The binding affinity of the WT and different variants of PhoSL toward the core fucose glycan substrate 1 as measured by microscale thermophoresis. Error bars denote the means ± SD in three independent assays. d) The electrostatic potential surfaces for both tryptophan and its electron‐rich derivative, 5‐methoxy‐tryptophan. Molecular surfaces with negative charges and positive charges are colored in red and blue, respectively. e) The CH–π contact trajectories of W28 in WT and 5‐methoxy‐tryptophan mutant (Mut, W28_mut_ in the text) PhoSL from MD simulations. f) The frequency distribution of the CH–π contacts of W28 in the WT and mutant PhoSL from two independent MD simulations for each system. g) The depiction of PhoSL‐glycan interactions. The interactions between PhoSL residues and the glycan residues are indicated by lines, and their thickness is linearly scaled with the frequency of the corresponding interaction in MD simulations.

To test this, we first obtained a series of electron‐rich Trp derivatives from commercial sources and chemical synthesis (**Figure** **2**a). We then used the genetic code expansion system to replace W28 of PhoSL with these derivatives, as previously reported.^[^ ^32^ ^]^ The genetic code expansion strategy utilizes orthogonal translation systems (OTSs) to introduce PTMs or their analogs in the form of noncanonical amino acids into proteins in a site‐specific manner. The codon corresponding to W28 was mutated to the amber suppressor codon TAG followed by co‐expression of the pyrrolysyl‐tRNA and pyrrolysyl‐tRNA synthetase pair in *E. coli* BL‐21 cells. Here, the pyrrolysyl‐based system was previously modified with components from the phenylalanine synthetase, allowing highly efficient incorporation of Trp derivatives in both prokaryotes and eukaryotes.^[^ ^33^ ^]^ The corresponding Trp derivative was added to induce protein expression. The resulting proteins were purified by GST tag fusion at the C‐terminus of PhoSL to obtain high homogeneity (Figure 2b). To verify the efficient incorporation of Trp derivatives, we performed site mapping of PhoSL substituted with 5‐methyl‐Trp or 5‐methoxy‐Trp by tandem mass spectrometry analysis (Figure S2a–c, Supporting Information). These protein variants were further analyzed to probe whether the incorporated Trp derivatives affected glycan‐protein interaction. Notably, the binding affinity of PhoSL variants with 5‐methoxy, 6‐methoxy, 7‐methoxy, or 6,7‐dimethoxy Trp for the glycan substrate increased by 2.7‐fold, 1.6‐fold, 1.8‐fold, or 2.1‐fold, respectively, compared to the WT PhoSL (Figure 2c). In contrast, the binding affinity of PhoSL variants with 5‐methyl, 6‐methyl, or 7‐methyl (mild electron‐rich derivatives) only changed slightly compared to the WT PhoSL (Figure S3, Supporting Information). These results indicate that the CH–π interactions between the glycan substrate and PhoSL can be enhanced by substituting with electron‐rich derivatives of W28.

To further investigate the molecular basis of increased recognition by the 5‐methoxy substitution, we performed density functional theory calculations to compare the electrostatic potential surfaces of Trp and 5‐methoxy Trp. The result clearly showed that the addition of an electron‐rich methoxy group boosts the indole ring's electrostatic potential (Figure 2d). Next, we performed MD simulations of PhoSL trimer with 5‐methoxy substituted W28 (W28_mut_) in complex with glycans and compared the results with that of the WT PhoSL‐glycan complex (Videos S1 and S2, Supporting Information). The MD trajectories showed that W28_mut_ indeed made the CH–π contacts more stable than the WT (Figure 2e). The substitution also increased the average number of CH–π contact of W28 from 1.6 to 1.9 (Figure 2f). In contrast to the WT, in which W28 interacted with both GlcNAc1 and GlcNAc2 of the glycan, W28_mut_ in the mutant protein mainly interacted with the core GlcNAc1 (Figure 2g). These results are consistent with the experimental binding data, suggesting that the 5‐methoxy substitution of W28 strengthens the CH–π interactions between the glycan substrate and PhoSL, resulting in a higher binding affinity.

### 2.3 W28‐substituted PhoSL increases sensitivity for detecting and imaging core fucosylated glycans in vitro and in vivo

**Figure 3:**
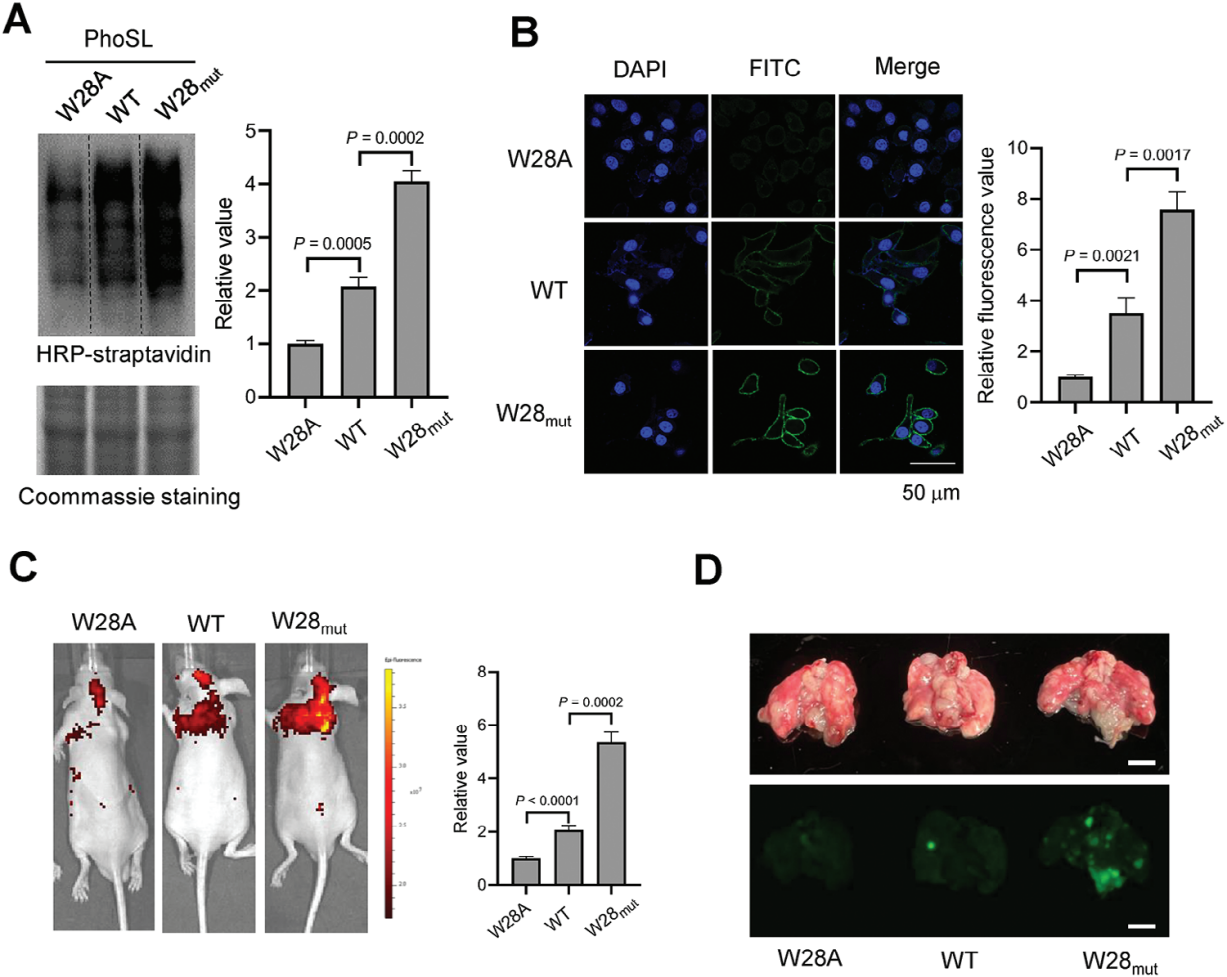
W28‐substituted PhoSL increases sensitivity for detecting and imaging core fucose glycans in vitro and in vivo. a) Western blotting and Coomassie analysis core fucosylated proteins of HepG2 cells by affinity pulldown with indicated PhoSL variants. b) Immunofluorescence analysis of core fucose glycans on HepG2 cell surface using indicated PhoSL variants. The relative values were determined by Image J (*n* =  3 independent assays). Scale bar: 50 µm. c) Fluorescence images of lung metastasis model mice after injection with FITC labeled PhoSL for 1 h. The relative fluorescence intensity was determined by Living Image (*n* =  3 independent assays). Scale bar: 0.5 cm. d) Representative *ex vivo* lung images of mice from each group using the fluorescence gel imaging system. Error bars in (a), (b), and (c) denote the mean ± SD. Statistical analyses were performed by unpaired two‐tailed Student's t‐tests.

Lectins have been used for detecting and imaging glycans. However, their relatively low binding affinity toward glycans has hampered the application.^[^ ^34^, ^35^ ^]^ With engineered PhoSL variants of higher binding affinity in hand, we aimed to explore the utility in glycan detection and imaging. The WT, W28_mut_, and W28A PhoSL were individually conjugated with biotin. Cell lysates from the human liver cancer cell line HepG2 were separated by SDS‐PAGE, transferred to nitrocellulose membranes, and incubated with biotinylated PhoSL variants. Subsequently, membranes were incubated with horseradish peroxidase (HRP)‐labeled streptavidin, and detected by chemiluminescence imaging. Notably, a significantly higher detection signal was observed with the W28_mut_ PhoSL, compared to the WT PhoSL (**Figure** **3**a). As a negative control, a much lower signal was shown with the W28A PhoSL.

Next, we explored the imaging of cell surface core fucosylated glycans in live cells. We conjugated PhoSL variants with FITC, and validated that PhoSL specifically recognizes core fucosylated glycans on HepG2 cell surface by competitively abolishing the fluorescent signal with core fucosylated glycan substrate **1** (Figure S4, Supporting Information). We observed that the W28_mut_ PhoSL produced the highest fluorescent signal, while the W28A PhoSL produced the weakest signal (Figure 3b). This is consistent with the results obtained from the Western–blotting analysis.

We further investigate the imaging of core fucose glycans in vivo. Core fucosylated glycans have been reported to be overexpressed in various types of tumors, which is closely related to tumor growth and metastasis.^[^ ^36^, ^37^ ^]^ Imaging of core fucosylated glycans in live animals has largely been unexplored. To do this, we injected 4T1 cells (the murine breast cancer cell line) into nude mice via the tail vein to establish the lung metastasis model.^[^ ^38^ ^]^ Two weeks after the injection, we injected the FITC‐conjugated WT, W28_mut_, or W28A PhoSL via the tail vein to image core fucosylated glycans present in pulmonary metastatic nodules. One hour after the injection, we anesthetized and imaged the mice by the small animal live fluorescence imaging system. As shown in Figure 3c, the W28_mut_ PhoSL injection resulted in a 2.5‐fold increase in the fluorescence signal compared to the WT PhoSL. The negative control, injection of the W28A PhoSL only showed the background fluorescent signal. After we euthanized and dissected the mice, we found many tumor nodules in the lung tissues. The fluorescent signals were mainly localized in metastatic nodules, indicating the specificity of PhoSL. Consistently, labeling with the W28_mut_ PhoSL produced the strongest signal compared to the WT or W28A PhoSL (Figure 3d). Taken together, these data suggest that the W28_mut_ PhoSL is a superior tool for the sensitive detection and imaging of core fucosylated glycans both in vitro and in vivo.

### 2.4 Spatial Profiling of Core Fucosylated Proteins and Interacting Partners with W28‐Substituted PhoSL

**Figure 4:**
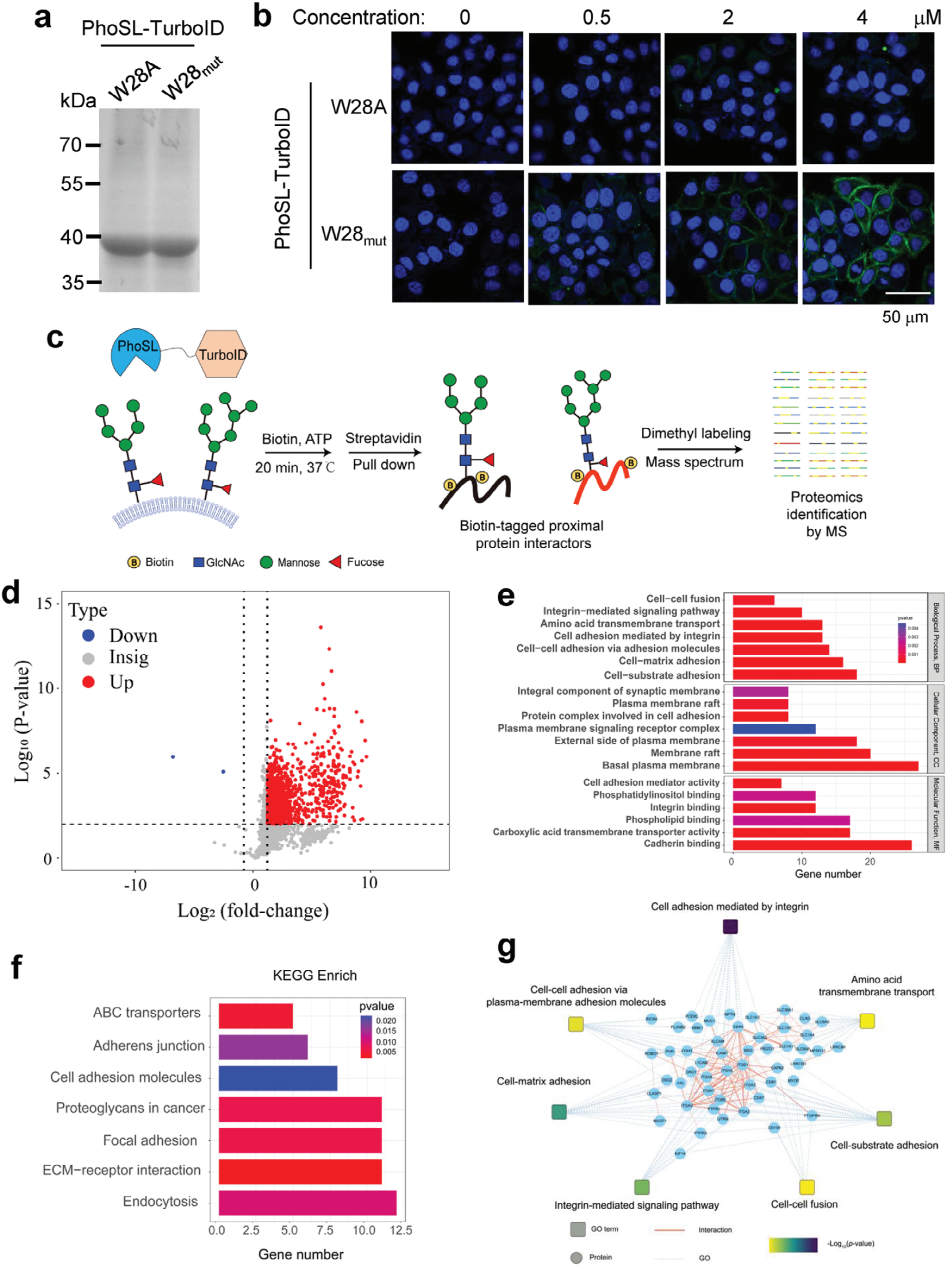
Spatial profiling of core fucosylated proteins and interacting partners with W28‐substituted PhoSL. a) Labelling and imaging of core‐fucosylated proteins and the interacting partners on HepG2 cells after incubation with various concentrations of W28A or W28_mut_ PhoSL‐TurboID. Scale bar: 50 µm. b) Coomassie brilliant blue staining of purified W28A or W28_mut_ PhoSL‐TurboID. c) Schematic diagram of proximity labeling and proteomics of core fucosylated proteins. d) Volcano plot for the identification of core fucosylation interactomes. Proteins with a fold change >5 and *p* value <0.01 were considered as core fucosylated proteins and the interacting partners and are highlighted in red. e) Gene ontology analysis of identified glycoproteins categorized by biological processes (GOBP), cellular components (GOCC), and molecular Function (GOMF). f) Kyoto Encyclopedia of Genes and Genomes (KEGG) pathway analysis of identified glycoproteins. g) Protein–protein interaction (PPI) network analysis of identified glycoproteins using the STRING database.

Core fucosylation has been demonstrated to play critical roles in both normal physiology and disease development.^[^ ^24^, ^25^ ^]^ Core fucosylation exerts the biological function not only by modifying protein substrates but also by coordinating with the neighboring interacting proteins. Thus, systemic profiling of core fucosylated proteins and the interacting partners will provide valuable insight into the underlying molecular mechanisms. We envision that proximity labeling strategies using an engineered biotin ligase (TurboID) could offer a powerful tool to profile core fucosylated proteins and the interacting network.^[^ ^39^ ^]^ In addition, our substituted PhoSL with stronger glycan recognition is expected to achieve a better performance and high signal‐to‐noise ratio in the proximity labeling. Thus, we genetically fused the biotin ligase TurboID with the W28_mut_ and W28A PhoSL to test their capability to identify core fucosylation interactomes in live cells (**Figure** **4**a). HepG2 cells were incubated with various concentrations of fusion proteins, and the biotinylation reaction was initiated by the addition of biotin and ATP and allowed to proceed for 30 min before quenching. Subsequent installation of streptavidin‐Fluor 488 showed that efficient labeling was achieved on the cell surface with the optimal concentration of the fusion W28_mut_ PhoSL at 4 µm (Figure 4b). In contrast, cell surface labeling with the W28A PhoSL only produced the background signal (Figure 4b).

Using the optimal labeling condition, we next performed enrichment of biotinylated proteins with streptavidin‐bound beads, on‐bead proteolytic digestion, and protein identification with liquid chromatography coupled mass spectrometry (LC‐MS) (Figure 4c). We compared cells treated with the W28_mut_ PhoSL‐TurboID and the W28A PhoSL‐TurboID to identify proteins predominantly mediated by the glycan‐binding effect. To achieve a high‐confidence assignment of identified proteins, the digested peptides were isotopically derivatized by NaCNBH_3_ or NaCNBD_3_ mediated dimethyl labeling before MS analysis.^[^ ^40^ ^]^ Only proteins with a fold change >5, *p*‐value <0.01 (Student's *t*‐test), and at least two identified unique peptides in at least two parallel tests were considered as core‐fucosylated proteins and the interacting partners. Using these criteria, we identified 451 proteins in HepG2 cells (Figure 4d; Table S1, Supporting Information). Among these proteins, 136 proteins were also identified as putative core‐fucosylated proteins in previous studies.^[^ ^41^, ^42^, ^43^ ^]^ Cellular localization analysis showed a majority (71.4%) of identified proteins were located on the plasma membrane. Gene ontology analysis of cellular components (GOCC) also confirmed that a large number of identified proteins were associated with the cellular membranes (Figure 4e). Gene ontology analysis of biological processes (GOBP) revealed that these identified proteins played critical roles in cell adhesion, integrin‐mediated signaling pathways, amino acid transmembrane transport, and cell–cell fusion (Figure 4e). Gene ontology analysis categorized by molecular Function (GOMF) further showed that the identified proteins were closely related to the binding of phosphatidylinositol, integrin, and cadherin (Figure 4e). The similar result was observed by enrichment analysis of the Kyoto Encyclopedia of Genes and Genomes (KEGG) pathway (Figure 4f). To further understand the cellular processes of the identified proteins, we constructed a protein–protein interaction (PPI) network using the STRING database and found that the most significantly enriched cluster was cell adhesion (Figure 4g). Together, these results accord well with the function of core‐fucosylation involved in cell–cell communications and provide useful information for further mechanistic understanding of core‐fucose‐mediated cellular processes.

### 2.5 A similar Strategy Applies to Increasing GafD Recognition of GlcNAc‐Containing Glycans

To demonstrate the broad applicability of our strategy for enhancing glycan‐protein recognition by optimizing CH–π interactions, we extended this approach to GafD, a lectin that specifically binds to *N*‐linked and *O*‐linked glycans with a terminal GlcNAc sugar.^[^ ^44^, ^45^ ^]^ Among the four Trp residues in GafD (W46, W85, W102, and W109), the previously solved crystal structure revealed that the latter three are solvent‐exposed and contribute to two putative glycan binding sites near W102 and W109 (Figure S5a, Supporting Information).^[^ ^46^ ^]^ To identify the sugar‐binding site, we performed atomistic MD simulations of GafD with GlcNAc sugars and observed numerous dynamic glycan binding events. These simulations revealed that W109 formed more stable CH–π interactions with GlcNAc compared to W102, identifying it as the optimal residue for modification (Figure S5b, Supporting Information).

**Figure 5:**
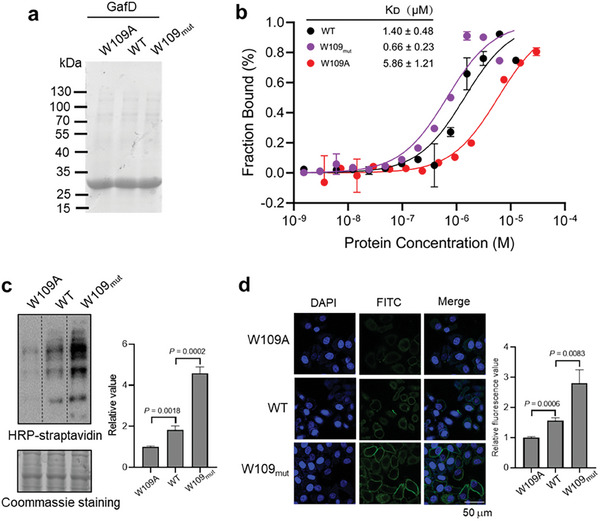
A similar strategy applies to increasing GafD recognition of GlcNAc‐containing glycans. a) Coomassie brilliant blue staining of purified W109A, WT, or W109_mut_ GafD. b) Thermophoretic analysis of the binding affinity of the indicated GafD variants with the FITC labeled GlcNAc glycan substrate (*n* =  3 independent assays). c) Western blotting and Coomassie analysis GlcNAc‐containing glycoproteins of HepG2 cells by affinity pulldown with indicated GafD variants. d) Immunofluorescence analysis of GlcNAc‐containing glycans on HepG2 cell surface using indicated GafD variants. The relative values were determined by Image J (*n* =  3 independent assays). Scale bar: 50 µm. Error bars in (b), (c), and (d) denote the mean ± SD. Statistical analyses were performed by unpaired two‐tailed Student's *t*‐tests.

Having identified W109 as the optimal site for enhancement, we hypothesized that replacing it with a more electron‐rich tryptophan derivative could strengthen favorable CH–π interactions with GlcNAc. Indeed, genetically incorporating 5‐methoxy Trp at W109 increased GafD's binding affinity for the GlcNAc glycan substrate by 2.3‐fold compared to the WT (K_D_ of 0.66 ± 0.23 µm vs 1.40 ± 0.48 µm). In contrast, the alanine mutant W109A exhibited a 4.2‐fold reduction in affinity (K_D_ of 5.86 ± 1.21 µm), highlighting the importance of W109 (**Figure** **5**a,b). Validation via Western‐blotting analysis of cell lysates and fluorescent imaging of live cells showed W109_mut_ yielded the strongest detection signal in both assays, indicating the enhanced glycan binding (Figure 5c,d). Tumor cells are coated with sialic acids at the termini of glycans. To rule out the effect of terminal sialic acids for glycan‐protein recognition, we used sialidases to remove sialic acids on the cell surface and detected the sialylation using a well‐established chemoenzymatic labeling strategy mediated by a specific glycosyltransferase CgtA.^[^ ^47^ ^]^ Treatment with sialidases almost abolished the fluorescence signal of sialylation, but had no apparent impact on the recognition of W109_mut_ for GlcNAc, indicating the binding between GafD and GlcNAc regardless of sialic acids (Figure S6a,b, Supporting Information). We also labeled and imaged the terminal GlcNAc moieties on the cell surface using GalT1^Y289L^ mediated chemoenzymatic labeling method coupled with bioorthogonal reactions.^[^ ^48^ ^]^ The result showed that the fluorescence signal detected by chemoenzymatic labeling strategy displayed a comparable level as that detected by W109_mut_ (Figure S6c, Supporting Information). Together, these results demonstrate the power and broad applicability of CH–π interaction enhancement through genetic code expansion as a generalizable approach to improve glycan‐protein recognition. This underscores the versatility of optimizing CH–π interactions for improving glycan recognition across diverse proteins.

## 3 Discussion

Glycans contain important biological information that is frequently decoded through specific interactions with glycan‐binding proteins (lectins). These interactions can trigger cellular signaling pathways, modulate cell–cell communications, and reprogram cell behaviors.^[^ ^4^, ^9^ ^]^ Recent studies have also highlighted links between aberrant glycan‐protein interactions and disease development/progression.^[^ ^49^, ^50^ ^]^ Given their glycan specificity, lectins have now been used as a valuable tool to probe glycan structure and image glycans. However, most known glycan‐protein interactions have relatively weak affinity, with K*_D_* values in the sub‐mM range.^[^ ^51^, ^52^ ^]^ This low binding affinity has hindered the application for glycan detection and imaging in cells. Here we develop a versatile approach to increase glycan‐protein interactions by genetically incorporating unnatural amino acids (electron‐rich Trp derivatives) into the glycan binding site. Single‐site incorporation significantly increased the binding affinity by two to three‐fold, enabling the detection and imaging of glycans with higher sensitivity. Since multivalency is known to enhance avidity between glycans and lectins,^[^ ^16^, ^20^ ^]^ multivalent presentation of these modified lectins could further improve glycan recognition. Moreover, because the genetic incorporation occurs inside cells, the enhanced recognition is poised for living cell imaging of glycan localization and trafficking. It may also provide opportunities to modulate cellular functions dependent on glycan recognition.

Current strategies for glycan detection and imaging consist of lectin binding, metabolic labeling, and chemoenzymatic labeling. These strategies vary in specificity, sensitivity, and applications, and are complementary to each other. Notably, while chemoenzymatic labeling has exceptional specificity and sensitivity in glycan detection, its translation to in vivo imaging remains unattainable. On the other hand, the metabolic labeling exhibits a broad range of in vitro and in vivo applications whereas the specificity of the labeling is unsatisfactory due to the intricate metabolic milieu within living systems. Our strategy, which engineers lectins to possess a higher binding affinity to glycans, not only achieves the detection sensitivity comparable to the chemoenzymatic labeling but also enables in vivo imaging of glycans. Therefore, it provides a promising tool for glycan imaging and detection both in vitro and in vivo.

CH–π interactions are a prominent feature of glycan‐protein recognition, being present not only in glycan‐lectin binding but also in the transition state stabilization during glycosyl‐transfer and glycosyl‐hydrolysis reactions.^[^ ^18^, ^20^ ^]^ In these reactions, the cationic or cationic‐like anomeric carbon is stabilized by the aromatic π orbitals in the transition state. Thus, our approach to strengthening CH–π contacts and enhancing glycan‐protein recognition through genetic incorporation of electron‐rich aromatic amino acids may have important implications for designing improved enzyme catalysts for glycan synthesis and probing reaction mechanisms. More broadly, electrostatic π interactions play central roles in biomolecular recognition underlying many critical biological processes.^[^ ^53^, ^54^, ^55^ ^]^ The ability to genetically manipulate the π system thus provides an unprecedented opportunity for advancing our understanding of biological recognition. Overall, this work establishes the CH–π interaction as a flexible target for modulating glycan‐protein binding and provides a platform to investigate the broader impacts of aromatic residues on glycan‐focused enzymes and biomolecular recognition.

## 4 Experimental Section

### Reagents

ATP (BD112724), 5‐methyl‐L‐tryptophan (BD38329), 6‐methyl‐L‐tryptophan (BD562299), 6‐methoxy‐L‐tryptophan (BD237659), 7‐methyl‐L‐tryptophan (BD00839661) and 7‐methoxy‐L‐tryptophan (BD562297) were purchased from Bidepharm Inc. The 5‐methoxy‐L‐tryptophan (M353041) was purchased from Aladdin. Biotin (A100340‐0001) was purchased from BBI Life Sciences. Streptavidin Agarose Resin (20353) was purchased from Thermo Scientific. Dylight 488 and streptavidin (YEASEN, 35103ES60) were used in at a dilution of 1:1000.

### Cell Culture

Cell lines 293T, 4T1and HepG2 were all obtained from American Type Cell Culture (ATCC). These cell lines were cultured in Dulbecco's Modified Eagle's Medium (DMEM) (Gibco) supplemented with 10% FBS (HyClone) and 1% Penicillin/Streptomycin in an incubator under 5% CO_2_ at 37 °C.

### Plasmid Construction

The plasmid pBK bearing the chPheRS, and the plasmid pNEG carrying the chPheT were provided by Prof. Shixian. Lin (Zhe Jiang University). The protein sequence of PhoSL (A0A384E107‐1) and GafD (Q47341) was obtained from the UniProt database and the gene sequences were synthesized by SunYa. PhoSL or GafD and GST tag were inserted into pNEG‐chPheT to generate pNEG‐chPheT‐PhoSL or GafD‐GST. Site‐directed mutagenesis of PhoSL and GafD was performed by PCR. Primers for site‐directed mutation study are listed below.

**Table:** 

Primers	Sequence 5’→3’
PhoSLW28AF	GGCAGGGCGGTGGCCCAGTGGGACACCAAC
PhoSLW28AF	GGCCACCGCCCTGCCGTCGCCGAAGTCCAG
PhoSLW28TAGF	GGCAGGTAGGTGGCCCAGTGGGACACCAAC
PhoSLW28TAGR	GGCCACCTACCTGCCGTCGCCGAAGTCCAG
GafDW109AF	CATGGGAGAATGTCTTTTCCGGAGCGTGCGTGG
GafDW109AR	ATACATAATTTCCCACGCACGCTCCGGAAAAGA
GafDW109TAGF	CATGGGAGAATGTCTTTTCCGGATAGTGCGTGG
GafDW109TAGR	ATACATAATTTCCCACGCACTATCCGGAAAAGA

### Protein Expression and Purification

For expression of wild type and W28A PhoSL, the corresponding plasmid pNEG was transformed into DH10B cells. At OD600 ≈0.8, 0.2% arabinose and 0.1 mM ZnCl_2_ were added into the medium to induce protein expression and sequentially cultured for 20 h at 16 °C. For PhoSL variants incorporated UAAs, the plasmid pNEG bearing PhoSL with an amber codon at the site of W28 and the plasmid pBK bearing corresponding chPheRS were co‐transformed into the DH10B cells in LB medium supplemented with 50 µg mL^−1^ kanamycin and 100 µg mL^−1^ ampicillin. At OD600 ≈0.8, 0.2% arabinose, 0.1 mm ZnCl_2_ and 2 mm corresponding tryptophan derivatives were added into medium to induce protein expression. After culture for 20 h at 16 °C, cells were then harvested by centrifugation at 10 000 *g* for 5 min and suspended in lysis buffer (20 mm Tris‐HCl, pH 7.4, 150 mm NaCl, and 2 mm β‐Me). The cells were broken by ultra‐sonication with 40% power for 10 min (3 sec on and 7 sec off) on ice and centrifuged at 15 000 *g* for 30 min to remove precipitants. The supernatants of lysates were incubated with GST‐tag purification resin (Beyotime) for 2 h at 4 °C and subsequently loaded onto an affinity chromatography column. After washing with lysis buffer, the proteins were eluted with the elution buffer (50 mM Tris‐HCl, 150 mm NaCl, 10 mm GSH, pH 8.0). Purified proteins were further concentrated using a 10 kD AmiconH Ultra Centrifugal Filter Unit (Millipore, Ireland), and then desalted with MST buffer (PBS buffer, 0.05% Tween‐20, pH 7.4). Proteins were then analyzed by 10% SDS‐PAGE. The protein concentration was determined by coomassie staining.

For the purification of GafD, the bacteria were collected and resuspended in 10 mL of PBS and sonicated thoroughly on ice. Then the lysate was diluted to 80 mL with PBS and centrifuged at 14 000 g for 30 min to discard the supernatant. The pellet was resuspended in PBS containing 25%(w/v) sucrose and centrifuged at 14 000 × g for 30 min to discard the supernatant. After resuspending with PBS containing 25% (w/v) sucrose for five times, the pellet was redissolved in 20 mL of 5 m guanidine hydrochloride (Gu‐HCl) with 0.3 m sodium sulfite at room temperature. 2 mL of 2‐nitro‐5‐(sulfothio)‐benzoate (NTSB) was added into reaction until the solution turned pale yellow. The protein was precipitated after the addition of 180 mL H_2_O and centrifuged at 10 000 × g for 10 min. Then the pellet was resuspended in 5 m guanidine hydrochloride (Gu‐HCI) with a concentration of 1 mg mL^−1^. The protein solution was diluted ten folds and dialyzed three times in the folding solution (0.7 m L‐arginine, 50 mm Tris‐HCl, 5 mm EDTA, 4 mm cysteamine, and 2 m cystamine, pH 8.0) at 4 °C. After dialysis, the precipitated protein was removed by centrifugation and purified proteins were obtained in the supernatant. The proteins were further concentrated, desalted, and analyzed by 10% SDS‐PAGE.

### Chemical Synthesis of 6,7‐Dimethoxy Trp (1)

 
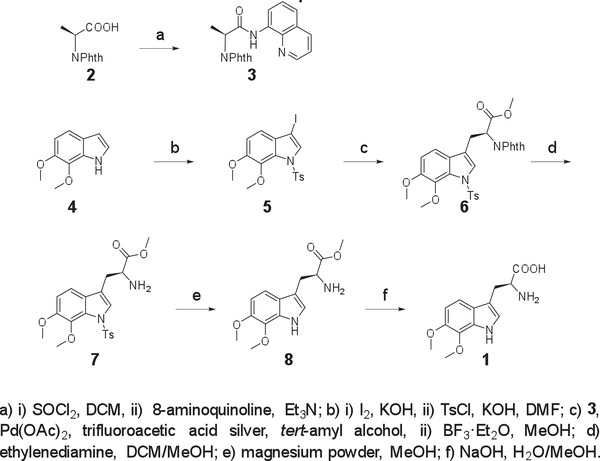


The Phth‐protected amino acid **2** (2.0 g, 9.1 mmol), thionyl chloride (3.3 mL, 45.5 mmol), and 3 drops of DMF in DCM (20 mL) were heated at 40 °C for 2 h. After completion of the reaction, DCM and excess of thionyl chloride were removed by rotary evaporation. The acid chloride intermediate was then dissolved in dry DCM (50 mL), and the solution was added slowly to a stirred solution of 8‐aminoquinoline (1.45 g, 10.0 mmol) and Et_3_N (1.52 mL, 11.0 mmol) in dry DCM (50 mL) at 0 °C. The mixture was stirred for 20 min, quenched by DCM (30 mL), and filtered. The collected solution was then washed by aqueous HCl (50 mL, 1 m), saturated NaHCO_3_ aqueous (50 mL), and brine (50 mL). The combined organic layer was dried over MgSO_4_, filtered, and concentrated in *vacuo*. The residue was purified by flash column chromatography on silica gel to afford compound **3** (2.80 g, 89%) as a white solid. ^1^H NMR (600 MHz, CDCl_3_) δ 10.32 (s, 1H), 8.72 (dd, *J* = 5.7, 3.3 Hz, 1H), 8.69 (dd, *J* = 4.2, 1.6 Hz, 1H), 8.14 (dd, *J* = 8.3, 1.6 Hz, 1H), 7.90 (dd, *J* = 5.4, 3.0 Hz, 2H), 7.76 (dd, *J* = 5.4, 3.1 Hz, 2H), 7.54–7.49 (m, 2H), 7.42 (dd, *J* = 8.3, 4.2 Hz, 1H), 5.27 (q, *J* = 7.3 Hz, 1H), 1.98 (d, *J* = 7.4 Hz, 3H).

To a solution of compound **4** (1.3 g,11.3 mmol) in DMF (22 mL) was added freshly powdered KOH (1.58 g, 28.2 mmol). The mixture was stirred for 30 min at room temperature. Then, a solution of iodine (3 g, 11.8 mmol) in DMF (22 mL) was added dropwise to the above solution. The resulting mixture was stirred for 1 h. Then, powdered KOH (1.58 g, 28.2 mmol) and TsCl (4.5 g, 23.7 mmol) were added to the mixture. After completion of the reaction, the resulting mixture was diluted with water (200 mL) and extracted with EtOAc (3 × 250 mL). The combined organic layer was washed with brine, dried over MgSO_4_, filtered, and concentrated in *vacuo*. The residue was purified by flash column chromatography on silica gel using petroleum/ethyl acetate to afford compound **5** (4.20 g, 81%) as a white solid. ^1^H NMR (600 MHz, CDCl_3_) δ 7.84 (s, 1H), 7.78 (d, *J* = 8.4 Hz, 2H), 7.25 (d, *J* = 8.1 Hz, 2H), 7.05 (d, *J* = 8.6 Hz, 1H), 6.96 (d, *J* = 8.6 Hz, 1H), 3.87 (s, 3H), 3.85 (s, 3H), 2.36 (s, 3H).

A pressure flask was charged with compound **3** (1.0 g, 2.90 mmol), compound **5** (2.0 g, 4.4 mmol), Pd(OAc)_2_ (65.0 mg, 0.3 mmol), and trifluoroacetic acid silver (890 mg, 4.0 mmol). The flask was flushed with argon. Then, degassed *tert*‐amyl alcohol (10.0 mL) was added, and the mixture was vigorously stirred at 60 °C for 16 h. After completion of the reaction, the mixture was diluted with DCM and filtered. The solvent was concentrated in *vacuo*, and the residue was purified by a silica gel column to afford an intermediate compound as a white solid. The intermediate compound was dissolved in MeOH (15 mL). Then, BF_3_·Et_2_O (3.65 mL, 29.6 mmol) was added, and the mixture was heated up to 100 °C for 24 h. After cooling to room temperature, saturated NaHCO_3_ aqueous was added to quench the reaction. The aqueous phase was washed by DCM three times. The combined organic layer was dried over MgSO_4_, filtered, and concentrated in *vacuo*. The residue was purified by flash column chromatography on silica gel (hexane/EtOAc = 2/1) to afford compound **6** (680 mg, 42%) as a yellow oil. ^1^H NMR (500 MHz, CDCl_3_) δ 7.82 (dd, *J* = 5.4, 3.1 Hz, 2H), 7.73 (dd, *J* = 5.5, 3.0 Hz, 2H), 7.54 (d, *J* = 8.4 Hz, 2H), 7.49 (s, 1H), 7.18 (d, *J* = 8.6 Hz, 1H), 7.08 (d, *J* = 8.2 Hz, 2H), 6.85 (d, *J* = 8.6 Hz, 1H), 5.23 (dd, *J* = 9.7, 6.2 Hz, 1H), 3.82 (s, 3H), 3.80 (s, 3H), 3.79 (s, 3H), 3.66–3.59 (m, 2H), 2.31 (s, 3H).

To a solution of compound **6** (500 mg, 0.9 mmol) in CH_2_Cl_2_ (2.5 mL) and MeOH (2.5 mL) was added ethylenediamine (600 µL, 9.0 mmol) in one portion. The mixture was stirred at room temperature for 2 h. The solvent was removed by rotary evaporation, and the residue was purified by flash column chromatography on silica gel (DCM/MeOH = 10:1) to give compound **7** (350 mg, 90%) as a yellow oil. ^1^H NMR (500 MHz, CDCl_3_) δ 7.75 (d, *J* = 8.4 Hz, 2H), 7.58 (s, 1H), 7.23 (d, *J* = 8.1 Hz, 2H), 7.18 (d, *J* = 8.6 Hz, 1H), 6.89 (d, *J* = 8.6 Hz, 1H), 3.85 (s, 3H), 3.83 (s, 3H), 3.83 – 3.80 (m, 1H), 3.73 (s, 3H), 3.14 (ddd, *J* = 14.4, 5.1, 0.8 Hz, 1H), 2.98 – 2.90 (m, 1H), 2.35 (s, 3H), 1.62 (s, 2H).

Magnesium powder (240 mg, 10 mmol) and compound **7** (216 mg, 0.5 mmol) were added to a round‐bottomed flask under the protection of argon. The anhydrous methanol was added to the bottle through a syringe, and the mixture was stirred vigorously at room temperature for 2 h. The solvent was removed by rotary evaporation, and the residue was purified by flash column chromatography on silica gel (DCM/MeOH = 10:1) to give compound **8** (116 mg, 84%) as a yellow oil. ^1^H NMR (400 MHz, CDCl_3_) δ 8.32 (s, 1H), 7.24 (d, *J* = 8.6 Hz, 1H), 6.98 (s, 1H), 6.84 (d, *J* = 8.6 Hz, 1H), 3.98 (s, 3H), 3.92 (s, 3H), 3.82 (dd, *J* = 7.2, 5.1 Hz, 1H), 3.71 (s, 3H), 3.23 (dd, *J* = 14.4, 4.6 Hz, 1H), 3.01 (dd, *J* = 14.4, 7.7 Hz, 1H), 1.83 (s, 2H).

Compound **8** (110 mg, 0.4 mmol) was dissolved in MeOH (10 mL), and NaOH (48 mg, 1.2 mmol) in H_2_O (10 mL) was added. The mixture was heated under reflux for 8 h. After completion of the reaction, the mixture was cooled to room temperature. Then, the solvent was removed by rotary evaporation, and the crude product was further purified by Bio‐Gel‐P2 (water) to give compound **1** (97 mg, 92%) as a white crystal. ^1^H NMR (500 MHz, D_2_O) δ 7.30 (d, *J* = 8.7 Hz, 1H), 7.21 (s, 1H), 6.94 (d, *J* = 8.7 Hz, 1H), 4.27 (dd, *J* = 7.3, 5.3 Hz, 1H), 3.89 (d, *J* = 1.5 Hz, 6H), 3.38 (dd, *J* = 15.4, 5.2 Hz, 1H), 3.29 (dd, *J* = 15.4, 7.4 Hz, 1H). ^13^C NMR (126 MHz, D_2_O) δ 171.86, 146.10, 133.30, 129.86, 124.75, 123.54, 113.73, 107.94, 106.75, 60.62, 57.15, 53.08, 25.41. HRMS (ESI) Calcd. for C_13_H_17_N_2_O_4_ [M+H]^+^ 265.1183; found, 265.1179.

### Detection of Trp Derivative‐Incorporated PhoSL Variants by LC‐MS

Purified PhoSL‐GST was freeze‐dried and resuspended in 50 mm NH_4_HCO_3_ (pH 8.0). The mixtures were digested by Glu‐C with shaking at 37 °C for 4 h, quenched by TFA acidification, desalted, and freeze‐dried for further LC‐MS analysis. Freeze‐dried samples were dissolved in solution A (0.1% formic acid in water) and trapped onto a homemade 150 µm × 20 mm C18 precolumn automatically by a Thermo EASY nLC 1200 system (Thermo Fisher Scientific). The bound peptides were then separated by a homemade 150 µm × 15 cm C18 analytical column over a 30 min gradient using solution A and solution B buffers (0.1% formic acid in 80% acetonitrile). For PRMs, the Orbitrap Fusion Lumos mass spectrometer (Thermo Fisher Scientific) was configured to collect MS/MS scans corresponding to several precursor targets in the Tryptophan28 (W28)‐containing sequence (Table S1, Supporting Information). Full MS was acquired from 300–1400 m z^−1^ (120 000 resolution, 50ms maximum inject time, 5e5 AGC target), and MS/MS was collected at a resolution of 15 000, maximum inject time of 30 ms, and AGC target of 5e4.The normalized collision energy of 30.

### Microscale Thermophoresis (MST)

Protein‐glycopeptide interactions were analyzed by microscale thermophoresis (MST). Glycopeptides were first labeled with fluorescein isothiocyanate (FITC). FITC ‐glycopeptide (50 nm) was mixed up with the gradient dilution of the protein in MST buffer (PBS buffer, 0.05% Tween‐20, pH 7.4). The samples were siphoned into the glass capillaries (Nano Temper Technologies, cat#MO‐K022) and MST was performed in an NT.115 Monolith instrument (Nano Temper Technologies, Munich, Germany) using a Blue LED with 20% blue LED excitation power, 40% IR laser power for excitation in three independent replicates at 25 °C. The dissociation constants (K_D_) were calculated by K_D_ model with a 1:1 stoichiometry per binding partner by MO Affinity Analysis Software. All data were processed by the GraphPad Prism 8 software.

### Molecular Dynamics Simulation

Initial models of the PhoSL and GafD proteins were built from their respective crystal structures (PDB IDs 6FX1 and 1OIO). For PhoSL, three core fucose N‐glycans were modeled as βDGlcNAc(1→2)αDMan(1→6)[βDGlcNAc(1→2)αDMan(1→3)]βDMan(1→4)βDGlcNAc(1→4)[αLFuc(1→6)]βDGlcNAc in the Glycan Reader Sequence format.^[^ ^56^ ^]^ For GafD, 0.1M GlcNAc sugars were added around the protein to sample potential binding sites. Both protein systems were solvated in cubic water boxes with 0.15 m NaCl to mimic physiological conditions using the CHARMM‐GUI server^[^ ^57^ ^]^ or Gromacs tools. The CHARMM36m force field^[^ ^58^ ^]^ with WYF π‐cation corrections^[^ ^59^ ^]^ was used to model the proteins, glycans, and ions. TIP3P water was used to solvate the systems. Initial glycan and 5‐methoxy‐tryptophan parameters were generated through the Glycosylation and Non‐standard Amino Acid modules in CHARMM‐GUI to ensure compatibility with CHARMM36m.

Neighbor searching was performed every 20 steps in the MD simulations. The PME algorithm was used for electrostatic interactions with a cut‐off of 1.2 nm. A reciprocal grid of 64 × 64 × 64 cells with 4th order B‐spline interpolation was used. A single cut‐off of 1.2 nm was used for Van der Waals interactions. The V‐rescale algorithm was used for temperature coupling. Temperature and pressure were kept constant at 300 K. All covalent bonds with hydrogen atoms of the protein and water molecules were constrained by the LINCS algorithm. Each system was minimized for 1000 steps, then equilibrated 1 ns in an NPT ensemble with position restraints on all heavy atoms of the complex. The hydrogen mass repartitioning technique was employed with a single LINCS iteration (expansion order 4), which allowed an integration time step of 4 fs to be used. The productive simulations were performed in NVT ensemble.

All MD simulations were performed using a GPU‐accelerated version of Gromacs 2021.5. Two 5000 ns production runs were carried out for the PhoSL systems. For GafD, two 1000 ns runs were performed. Molecular structures were visualized using Pymol and protein–glycan interactions analyzed with GetContacts (https://getcontacts.github.io/) and PLUMED^[^ ^60^ ^]^ and visualized using R. To specifically analyze CH–π interactions, the coordination number between hydrogen atoms of glycan CH groups and the center of aromatic rings for aromatic residues was calculated. Note that Trp has an extra five‐member ring, different from Tyr and Phe which only have one six‐member ring. A switching function was used as follows to define the CH–π coordination number:

(1) $$c\;=\frac{1-{\left(\frac{r_{ij}}{r_{0}}\right)}^{n}}{1-{\left(\frac{r_{ij}}{r_{0}}\right)}^{m}}$$

where *c* is 1 if the contact between hydrogen atom *i* and the ring center *j* is formed, or 0 otherwise. The cutoff *r*_0_ was set to 0.3 nm. The exponents n and m were set to 6 and 12, respectively.

To explore the effects of replacing tryptophan residues with electron‐rich derivatives on the indole ring's electrostatic potential, Density Functional Theory (DFT) quantum mechanical calculations were conducted using the Gaussian16 software with the B2LYP functional and 6–311G+(2d,p) basis set. Visualization of the electrostatic potential surfaces was achieved using GaussianView 6.

### Constructions of FITC or Biotin‐Conjugated PhoSL and GafD

FITC or biotin‐coupled PhoSL and GafD were produced using FITC conjugation kit (Sangon Biotech, D601049) and a Biotin conjugation kit (Sangon Biotech, D601048), respectively. Briefly, PhoSL or GafD proteins were desalted in PBS and incubated with FITC or activated biotin at 37 °C for 90 min. The coupling proteins with high purity can be obtained by desalting column centrifugation.

### Western Blotting Analysis

Cells were lysed in RIPA buffer (50 mm Tris (pH 7.4), 150 mm NaCl, 1% NP‐40, 0.5% sodium deoxycholate, 0.1% SDS) containing protease inhibitor cocktail (Roche). The protein concentration was determined by BCA Protein Assay Kit (Beyotime). Approximately 50 µg of protein lysate was resolved on a 10% SDS‐PAGE gel, transferred to a nitrocellulose membrane, and incubated with Biotin‐PhoSL at 4 °C overnight. After washing with PBST buffer for three times, the membrane was incubated with HRP‐labeled Streptavidin (1:5000) for 1 h and detected by Tanon 5200 chemiluminescence imaging analysis system. The intensity of protein bands was quantified using Image J.

### Detection of Cell‐Surface Core Fucose on Living Cells by Fluorescence Microscopy

Fut8 overexpressing HepG2 cells were seeded and cultured into coverslip for 24 h. After washing three times with PBS, cells were incubated with FITC‐PhoSL in the labeling buffer (1% FBS, 10 mM HEPES pH 7.9 in PBS) for 2 h at 37 °C. Cells were washed three times with PBS, and fixed with 4% paraformaldehyde. Then cell nuclei were stained with DAPI in PBS for 30 min at 25 °C. Coverslips were washed three times with PBS and plated into a glass slide. The cells were imaging by using a laser scanning confocal microscope (FV3000).

### Chemoenzymatic Labeling of Sialylation and Terminal GlcNAc Moieties Glycoproteins on Cell Surface

HepG2 cells were seeded and cultured into a coverslip for 24 h. After washing three times with reaction buffer (PBS containing 3% FBS), cells were incubated with sialidase (1 U uL^−1^) in reaction buffer for 30 min at 37 °C to remove sialic acids. The reactions with the absence of sialidase were performed as parallel. For the labeling reaction of sialylation, 50 um of UDP‐GalNAz, 10 mm Mg^2+^ and 20 ug mL^−1^ CgtA were added into the labeling buffer at 37 °C for 1 h. For labeling of terminal GlcNAc moieties, cells were incubated with UDP‐GalNAz (100 µM) and GalT1 (20 ug mL^−1^) in the labeling buffer (1% FBS, 10 mM HEPES pH 7.9 in PBS) for 2 h at 37 °C. After washing three times with the labeling buffer, the cells were incubated with 30 µm DBCO‐PEG4‐biotin at 25 °C for 1 h. Followed by washing three times with labeling buffer, cells were incubated with streptavidin‐Alexa Fluor 488 in PBS containing 1% BSA for 30 min at 25 °C. Cells were washed three times with PBS, and fixed with 4% paraformaldehyde for 10 min. The nucleus was labeled with DAPI in PBS for 20 min at 25 °C. Coverslips were washed three times with PBS and plated into a glass slide. The cells were imaged by using a laser scanning confocal microscope (FV3000).

### Imaging of Core Fucosylation in Mouse Lung Metastasis Model

Mice experiments were authorized by the Institutional Animal Care and Use Committee of Zhejiang University. For tail vein injection, single‐cell suspensions of Fut8 overexpressing 4T1 cells (1 × 10^5^/100 µL PBS) were injected into the tail vein of 6‐week‐old nude mice. After 6 weeks, FITC‐PhoSL (20 mg kg^−1^) was injected into the tail vein of mice. After incubation for 60 min, the mice were placed in a small animal live imaging device for fluorescence intensity detection. Then the mice were euthanized and the lungs were removed for imaging in a gel imaging system with an excitation wavelength of 488 nm.

### Proximity Labeling Of Core Fucosylated Proteins and LC‐MS Analysis

A density of 5 × 10^6^ Fut8 overexpressing HepG2 cells per well was seeded into 6 cm^3^ culture dish. After 12 h, cells were washed three times with PBS and incubated with 1 mm Biotin, 1.5 mm ATP, and 2 µm TurboID‐PhoSL‐W28‐5MeOW in PBS buffer for 30 min at 37 °C. TurboID‐PhoSL‐W28A was used for negative control. Then, cells were washed three times with PBS and subsequently were lysed in RIPA buffer. The protein concentration was determined by BCA Protein Assay Kit. Approximately 1 mg of protein lysate was incubated with streptavidin beads (Pierce) with a rotation at 4 °C overnight. The beads were washed five times with PBS and reacted with 10 mm DTT in 50 mM NH_4_HCO_3_ (pH 8.0) at 56 °C for 1 h, and subjected to cysteine alkylation with 20 mm IAA at 25 °C for 45 min in the dark. Trypsin was added to digest proteins on beads overnight at 37 °C and quenched by adding TFA to a final concentration of 0.1%. The digested peptides were subsequently desalted using reverse phase C18 tips. and. Next, the peptides were freeze‐dried and then resuspended in 100 mm TEAB buffer (pH 8.5) for isotopic dimethyl labeling. Stable isotopic dimethyl labeling was conducted as previously described for quantitative comparison between the experimental groups of the peptides and the control groups.^[^ ^61^ ^]^ Briefly, the experimental groups (200 µL each) were treated with 8 µL of 4% (vol/vol) CD_2_O (Sigma), while the control groups (200 µL each) were treated with 8 µL of 4% (vol/vol) CH_2_O (Sigma). The solutions of the experimental and control groups were both incubated with 8 µL 0.6 m NaBH_3_CN (Sigma) on a shaker at room temperature for 1 h, followed by the addition of 32 µL of 1% (vol/vol) ammonia solution. After adding 16 µL of formic acid, the corresponding medium (CD_2_O + NaBH_3_CN) and light (CH_2_O + NaBH_3_CN) isotopically labeled experimental and control samples were mixed and then subjected to StageTip C18 desalting before MS analysis. The FASP‐digested and dimethyl‐labeled peptides were vacuum‐dried and redissolved in 0.1% FA. The samples were separated by a homemade 15 cm length reversed‐phase column (150 µm id) packed with Ultimate XB‐C18 1.9 µm resin (Welch materials). An Easy nLC 1200 system (Thermo) was used to fractionalize the peptides at a flow rate of 600 nL min^−1^ according to the following gradient: 7–12% B for 6 min, 12–30% B for 51 min, 30–45% B for 10 min, 45–95% B for 1 min, and 95% B for 7 min (solvent A was 0.1% formic acid, solvent B was 0.1% formic acid in 80% acetonitrile). The LC was coupled to an Orbitrap Fusion Tribrid mass spectrometer (Thermo) via a nanoelectrospray ionization source. Full‐scan mass spectra were acquired in the Orbitrap (scan range 300–1400 m z^−1^, 120 000 resolution, maximum injection time 100 ms and AGC target value of 5e5) in data‐dependent acquisition mode, followed by Higher‐energy Collision Dissociation (HCD) with 32% normalized collision energy. The ion trap was used to acquire MS2 detection with the top 20 MS/MS scans using higher‐energy collision dissociation (HCD) at 32% normalized collision energy. The AGC target was set to 5e3, and the maximum injection time was 35 ms. The target ions selected for MS/MS were dynamically exclusion within 18 s.

### Analysis of MS Data

The MS raw files were searched using MaxQuant (version 2.4.2.0) against the UniProt database (release on 2022, 20376 entry). The search parameter digestion enzyme was set as trypsin allowing a maximum of two missed tryptic cleavages, with the minimal peptide length as six amino acids. Carbamidomethyl cysteine was selected as a constant modification, while methionine oxidation and acetyl N‐terminal were allowed as variable modifications. For peptide identification, the mass tolerances for precursor ions and fragment ions were set to 20 ppm and 0.5 Da, respectively. A threshold of ≤1% was allowed for both the peptide false discovery rate (FDR) and protein FDR. Imputation of missing values was performed by deterministic minimum imputation strategy in each dataset.^[^ ^62^, ^63^, ^64^ ^]^ For filtering, “Perseus” software and Student's *t*‐test were used, and 1% FDR was applied. Proteins that were considered as significant were identified based on the following criteria: a minimum of two unique peptides in at least two parallel tests, a fold change of five or greater, and *p* < 0.01 in the experimental groups compared with the control groups. GO and pathway analyses were conducted using R language. The quantitative MS data have been deposited to the iProX (https://www.iprox.cn/page/PSV023.html;?url = 1690361810886oKRL) Password: sNnP

### Statistical Analysis

All experiments were performed at least three times. Error bars denote the mean ± SD. Statistical analyses were performed by unpaired two‐tailed Student's *t*‐test. Values of *p* < 0.05 were considered statistically significant.

## Conflict of Interest

The authors declare no conflict of interest.

## Author Contributions

Q.Z. and D.G. contributed equally to this work. W.Y. conceived the project, and designed cell biology and biochemistry experiments. Y.W. designed in silico experiments. Q.Z., D.G., J.H., and J.L. performed cell biology, biochemistry, and xenograft experiments. J.Z., N.H., X.K.C., and Y.W. performed molecular dynamics simulations and quantum mechanical calculations. Z.F. and W.Q. performed mass spectrometry analysis. Q.Z., W.Q., Y.W., and W.Y. analyzed the data. Q.Z., Y.W., and W.Y. wrote the paper with inputs from all authors.

## Supporting information

Supporting Information

Supplemental Video 1

Supplemental Video 2

## Data Availability

The data that support the findings of this study are available in the supplementary material of this article.

## References

[1] I. Alves , M. M. Vicente , A. M. Dias , J. Gaifem , C. Rodrigues , A. Campar , S. S. Pinho , Adv. Exp. Med. Biol. 2021, 1325, 265. 34495540 10.1007/978-3-030-70115-4_13

[2] S. T. Laughlin , J. M. Baskin , S. L. Amacher , C. R. Bertozzi , Science 2008, 320, 664. 18451302 10.1126/science.1155106 PMC2701225

[3] M. S. Macauley , P. R. Crocker , J. C. Paulson , Nat. Rev. Immunol. 2014, 14, 653. 25234143 10.1038/nri3737 PMC4191907

[4] R. Kleene , M. Schachner , Nat. Rev. Neurosci. 2004, 5, 195. 14976519 10.1038/nrn1349

[5] M. de Graaf , R. A. Fouchier , EMBO J. 2014, 33, 823. 24668228 10.1002/embj.201387442 PMC4194109

[6] M. Cohen , N. Hurtado‐Ziola , A. Varki , Blood 2009, 114, 3668. 19704115 10.1182/blood-2009-06-227041 PMC2766682

[7] P. C. Pang , P. C. Chiu , C. L. Lee , L. Y. Chang , M. Panico , H. R. Morris , S. M. Haslam , K. H. Khoo , G. F. Clark , W. S. Yeung , A. Dell , Science 2011, 333, 1761. 21852454 10.1126/science.1207438

[8] G. A. Rabinovich , M. A. Toscano , Nat. Rev. Immunol. 2009, 9, 338. 19365409 10.1038/nri2536

[9] N. C. Henderson , A. C. Mackinnon , S. L. Farnworth , F. Poirier , F. P. Russo , J. P. Iredale , C. Haslett , K. J. Simpson , T. Sethi , Proc. Natl. Acad. Sci. U S A 2006, 103, 5060. 16549783 10.1073/pnas.0511167103 PMC1458794

[10] L. Diaz‐Alvarez , E. Ortega , Mediat. Inflamm. 2017, 2017, 9247574. 10.1155/2017/9247574 PMC5457773 28607536

[11] K. Yamasaki , T. Kubota , T. Yamasaki , I. Nagashima , H. Shimizu , R. I. Terada , H. Nishigami , J. Kang , M. Tateno , H. Tateno , Glycobiology 2019, 29, 576. 30913288 10.1093/glycob/cwz025

[12] F. A. Quiocho , Annu. Rev. Biochem. 1986, 55, 287. 3527044 10.1146/annurev.bi.55.070186.001443

[13] S. Wisnovsky , C. R. Bertozzi , Curr. Opin. Struct. Biol. 2022, 75, 102395. 35653954 10.1016/j.sbi.2022.102395 PMC9811956

[14] L. Montalvillo‐Jimenez , A. G. Santana , F. Corzana , G. Jimenez‐Oses , J. Jimenez‐Barbero , A. M. Gomez , J. L. Asensio , J. Am. Chem. Soc. 2019, 141, 13372. 31390207 10.1021/jacs.9b03285

[15] Z. R. Laughrey , S. E. Kiehna , A. J. Riemen , M. L. Waters , J. Am. Chem. Soc. 2008, 130, 14625. 18844354 10.1021/ja803960x PMC2649776

[16] L. L. Kiessling , R. C. Diehl , ACS Chem. Biol. 2021, 16, 1884. 34615357 10.1021/acschembio.1c00413 PMC9004545

[17] J. L. Asensio , A. Ardá , F. J. Cañada , J. Jiménez‐Barbero , J. Acc. Chem. Res. 2013,16, 946. 10.1021/ar300024d 22704792

[18] C. H. Hsu , S. Park , D. E. Mortenson , B. L. Foley , X. Wang , R. J. Woods , D. A. Case , E. T. Powers , C. H. Wong , H. J. Dyson , J. W. Kelly , J. Am. Chem. Soc. 2016, 138, 7636. 27249581 10.1021/jacs.6b02879 PMC4924591

[19] J. Houser , S. Kozmon , D. Mishra , Z. Hammerova , M. Wimmerova , J. Koca , Chemistry 2020, 26, 10769. 32208534 10.1002/chem.202000593

[20] K. L. Hudson , G. J. Bartlett , R. C. Diehl , J. Agirre , T. Gallagher , L. L. Kiessling , D. N. Woolfson , J. Am. Chem. Soc. 2015, 137, 15152. 26561965 10.1021/jacs.5b08424 PMC4676033

[21] W. Chen , S. Enck , J. L. Price , D. L. Powers , E. T. Powers , C. H. Wong , H. J. Dyson , J. W. Kelly , J. Am. Chem. Soc. 2013, 135, 9877. 23742246 10.1021/ja4040472 PMC3715148

[22] M. I. Chavez , C. Andreu , P. Vidal , N. Aboitiz , F. Freire , P. Groves , J. L. Asensio , G. Asensio , M. Muraki , F. J. Canada , J. Jimenez‐Barbero , Chemistry 2005, 11, 7060. 16220560 10.1002/chem.200500367

[23] M. del Carmen Fernandez‐Alonso , F. J. Canada , J. Jimenez‐Barbero , G. Cuevas , J. Am. Chem. Soc. 2005, 127, 7379. 15898786 10.1021/ja051020+

[24] K. Bastian , E. Scott , D. J. Elliott , J. Munkley , Int. J. Mol. Sci. 2021, 22, 455. 33466384 10.3390/ijms22010455 PMC7795606

[25] J. Golay , A. E. Andrea , I. Cattaneo , Front. Immunol. 2022, 13, 929895. 35844552 10.3389/fimmu.2022.929895 PMC9279668

[26] T. Yokobori , S. Yazawa , T. Asao , N. Nakazawa , K. Shirabe , Sci. Rep. 2019, 9, 14503. 31601857 10.1038/s41598-019-51021-2 PMC6787216

[27] S. Hashimoto , T. Asao , J. Takahashi , Y. Yagihashi , T. Nishimura , A. R. Saniabadi , D. C. Poland , W. van Dijk , H. Kuwano , N. Kochibe , S. Yazawa , Cancer 2004, 101, 2825. 15536618 10.1002/cncr.20713

[28] K. Fujita , K. Hatano , E. Tomiyama , Y. Hayashi , M. Matsushita , M. Tsuchiya , T. Yoshikawa , M. Date , E. Miyoshi , N. Nonomura , Int. J. Cancer 2021, 148, 3111. 33594666 10.1002/ijc.33517

[29] A. Cabanettes , L. Perkams , C. Spies , C. Unverzagt , A. Varrot , Angew. Chem. Int. Ed. Engl. 2018, 57, 10178. 29956878 10.1002/anie.201805165

[30] Y. Kobayashi , H. Tateno , H. Dohra , K. Moriwaki , E. Miyoshi , J. Hirabayashi , H. Kawagishi , J. Biol. Chem. 2012, 287, 33973. 22872641 10.1074/jbc.M111.327692 PMC3464508

[31] L. L. Duan , T. Zhu , Y. Mei , Q. G. Zhang , B. Tang , J. Z. Zhang , J. Mol. Model. 2013, 19, 2605. 23479281 10.1007/s00894-013-1798-8

[32] H. Zhao , C. Liu , W. Ding , L. Tang , Y. Fang , Y. Chen , L. Hu , Y. Yuan , D. Fang , S. Lin , J. Am. Chem. Soc. 2022, 144, 6742. 35380832 10.1021/jacs.1c12944

[33] W. L. Ding , H. X. Zhao , Y. L. Chen , B. Zhang , Y. Yang , J. Zang , J. Wu , S. Lin , Nat. Commun. 2020, 11, 3154 32572025 10.1038/s41467-020-16898-y PMC7308279

[34] W. I. Weis , K. Drickamer , Annu. Rev. Biochem. 1996, 65, 441. 8811186 10.1146/annurev.bi.65.070196.002301

[35] S. Irumagawa , K. Hiemori , S. Saito , H. Tateno , R. Arai , Int. J. Mol. Sci. 2022, 23, 676. 35054861 10.3390/ijms23020676 PMC8775495

[36] C. F. Tu , M. Y. Wu , Y. C. Lin , R. Kannagi , R. B. Yang , Breast Cancer Res. 2017, 19, 111. 28982386 10.1186/s13058-017-0904-8 PMC5629780

[37] Y. C. Liu , H. Y. Yen , C. Y. Chen , C. H. Chen , P. F. Cheng , Y. H. Juan , C. H. Chen , K. H. Khoo , C. J. Yu , P. C. Yang , T. L. Hsu , C. H. Wong , Proc. Natl. Acad. Sci. U S A 2011, 108, 11332. 21709263 10.1073/pnas.1107385108 PMC3136320

[38] S. Yang , J. J. Zhang , X. Y. Huang , Methods Mol Biol 2012, 928, 221. 22956145 10.1007/978-1-62703-008-3_17 PMC3674868

[39] K. F. Cho , T. C. Branon , N. D. Udeshi , S. A. Myers , S. A. Carr , A. Y. Ting , Nat. Protoc. 2020, 15, 3971. 33139955 10.1038/s41596-020-0399-0

[40] N. Khidekel , S. B. Ficarro , P. M. Clark , M. C. Bryan , D. L. Swaney , J. E. Rexach , Y. E. Sun , J. J. Coon , E. C. Peters , L. C. Hsieh‐Wilson , Nat. Chem. Biol. 2007, 3, 339. 17496889 10.1038/nchembio881

[41] L. Cao , T. M. Lih , Y. Hu , M. Schnaubelt , S. Y. Chen , Y. Zhou , C. Guo , M. Dong , W. Yang , R. V. Eguez , L. Chen , D. J. Clark , A. Sodhi , Q. K. Li , H. Zhang , Nat. Commun. 2022, 13, 3910. 35798744 10.1038/s41467-022-31472-4 PMC9262967

[42] L. Jia , J. Li , P. Li , D. Liu , J. Li , J. Shen , B. Zhu , C. Ma , T. Zhao , R. Lan , L. Dang , W. Li , S. Sun , Theranostics 2021, 11, 6905. 34093861 10.7150/thno.56882 PMC8171077

[43] Y. Luo , Y. Wang , Y. Tian , H. Zhou , L. Wen , J. Am. Chem. Soc. 2023, 145, 15879. 37340703 10.1021/jacs.3c02976

[44] S. Saarela , S. Taira , E. L. Nurmiaho‐Lassila , A. Makkonen , M. Rhen , J. Bacteriol. 1995, 177, 1477. 7883703 10.1128/jb.177.6.1477-1484.1995 PMC176762

[45] Y. Liu , Z. M. Nelson , A. Reda , C. Fehl , ACS Chem. Biol. 2022, 17, 2153. 35819414 10.1021/acschembio.2c00282 PMC9391317

[46] M. C. Merckel , J. Tanskanen , S. Edelman , B. Westerlund‐Wikstrom , T. K. Korhonen , A. Goldman , J. Mol. Biol. 2003, 331, 897. 12909017 10.1016/s0022-2836(03)00841-6

[47] L. Wen , Y. Zheng , K. Jiang , M. Zhang , S. M. Kondengaden , S. Li , K. Huang , J. Li , J. Song , P. G. Wang , J. Am. Chem. Soc. 2016, 138, 11473. 27554522 10.1021/jacs.6b07132

[48] P. M. Clark , J. F. Dweck , D. E. Mason , C. R. Hart , S. B. Buck , E. C. Peters , B. J. Agnew , L. C. Hsieh‐Wilson , J. Am. Chem. Soc. 2008, 130, 11576. 18683930 10.1021/ja8030467 PMC2649877

[49] A. W. Chiang , S. Li , P. N. Spahn , A. Richelle , C. C. Kuo , M. Samoudi , N. E. Lewis , Curr. Opin. Struct. Biol. 2016, 40, 104. 27639240 10.1016/j.sbi.2016.08.008 PMC5161599

[50] T. D. Mubaiwa , E. A. Semchenko , L. E. Hartley‐Tassell , C. J. Day , M. P. Jennings , K. L. Seib , Pathog Dis. 2017, 75, ftx063. 28633281 10.1093/femspd/ftx063 PMC5808653

[51] Y. Ji , R. J. Woods , Adv. Exp. Med. Biol. 2018, 1104, 259. 30484253 10.1007/978-981-13-2158-0_13

[52] J. L. Asensio , F. J. Canada , M. Bruix , A. Rodriguez‐Romero , J. Jimenez‐Barbero , Eur. J. Biochem. 1995, 230, 621. 7607237 10.1111/j.1432-1033.1995.tb20604.x

[53] M. A. Gebbie , W. Wei , A. M. Schrader , T. R. Cristiani , H. A. Dobbs , M. Idso , B. F. Chmelka , J. H. Waite , J. N. Israelachvili , Nat. Chem. 2017, 9, 473. 28430190 10.1038/nchem.2720

[54] A. S. Mahadevi , G. N. Sastry , Chem. Rev. 2013, 113, 2100. 23145968 10.1021/cr300222d

[55] S. Qamar , G. Wang , S. J. Randle , F. S. Ruggeri , J. A. Varela , J. Q. Lin , E. C. Phillips , A. Miyashita , D. Williams , F. Strohl , W. Meadows , R. Ferry , V. J. Dardov , G. G. Tartaglia , L. A. Farrer , G. S. Kaminski Schierle , C. F. Kaminski , C. E. Holt , P. E. Fraser , G. Schmitt‐Ulms , D. Klenerman , T. Knowles , M. Vendruscolo , P. St George‐Hyslop , Cell 2018, 173, 720. 29677515 10.1016/j.cell.2018.03.056 PMC5927716

[56] S. J. Park , J. Lee , D. S. Patel , H. Ma , H. S. Lee , S. Jo , W. Im , Bioinformatics 2017, 33, 3051. 28582506 10.1093/bioinformatics/btx358 PMC5870669

[57] S. Jo , T. Kim , V. G. Iyer , W. Im , J. Comput. Chem. 2008, 29, 1859. 18351591 10.1002/jcc.20945

[58] J. Huang , S. Rauscher , G. Nawrocki , T. Ran , M. Feig , B. L. de Groot , H. Grubmüller , A. D. MacKerell Jr. , Nat. Methods 2017, 14, 71. 27819658 10.1038/nmeth.4067 PMC5199616

[59] H. M. Khan , A. D. MacKerell Jr. , N. Reuter , J. Chem. Theory Comput. 2019, 15, 7. 30562013 10.1021/acs.jctc.8b00839 PMC6467778

[60] P. Consortium , Nat. Methods 2019, 16, 670.31363226

[61] P. J. Boersema , R. Raijmakers , S. Lemeer , S. Mohammed , A. J. Heck , Nat. Protoc. 2009, 4, 484. 19300442 10.1038/nprot.2009.21

[62] B. J. Webb‐Robertson , H. K. Wiberg , M. M. Matzke , J. N. Brown , J. Wang , J. E. McDermott , R. D. Smith , K. D. Rodland , T. O. Metz , J. G. Pounds , K. M. Waters , J. Proteome Res. 2015, 14, 1993. 25855118 10.1021/pr501138h PMC4776766

[63] C. Lazar , L. Gatto , M. Ferro , C. Bruley , T. Burger , J. Proteome Res. 2016, 15, 1116. 26906401 10.1021/acs.jproteome.5b00981

[64] Y. Jiang , A. Sun , Y. Zhao , W. Ying , H. Sun , X. Yang , B. Xing , W. Sun , L. Ren , B. Hu , C. Li , L. Zhang , G. Qin , M. Zhang , N. Chen , M. Zhang , Y. Huang , J. Zhou , Y. Zhao , M. Liu , X. Zhu , Y. Qiu , Y. Sun , C. Huang , M. Yan , M. Wang , W. Liu , F. Tian , H. Xu , J. Zhou , et al., Nature 2019, 567, 257. 30814741 10.1038/s41586-019-0987-8

