# Supplementary material for: A Computational and Chemical Design Strategy for Manipulating Glycan‐Protein Recognition
Source: Adv Sci (Weinh). 2024 Apr 6;11(24):2308522. doi: 10.1002/advs.202308522 (PMC11199974; doi:10.1002/advs.202308522)
Supplement: Supplementary file 1 — Supporting Information [file ADVS-11-2308522-s002.pdf]

ADVANCED SCIENCE
Open Access

Supporting Information

for *Adv. Sci.*, DOI 10.1002/advs.202308522

A Computational and Chemical Design Strategy for Manipulating Glycan-Protein Recognition

Qiang Zhu, Didi Geng, Jingchao Li, Jinqiu Zhang, Haofan Sun, Zhiya Fan, Jiahui He, Ninghui Hao, Yinping Tian, Liuqing Wen, Tiehai Li, Weijie Qin, Xiakun Chu, Yong Wang and Wen Yi**

Supporting Information

A Computational and Chemical Design Strategy for Manipulating Glycan-Protein Recognition

Qiang Zhu, Didi Geng, Jingchao Li, Jinqiu Zhang, Haofan Sun, Zhiya Fan, Jiahui He, Ninghui Hao, Yinping Tian, Liuqing Wen, Tiehai Li, Weijie Qin, Xiakun Chu, Yong Wang, and Wen Yi**

Supplementary material

Supplementary Video legends

Supplementary Video 1: The 5-microsecond molecular dynamics simulation trajectory of PhoSL in complex with core fucose N-glycans. The three PhoSL protein monomers are depicted using ribbons colored green, cyan, and magenta. The core fucose N-glycan residues are shown as sticks with varying colors.

Supplementary Video 2: The 5-microsecond molecular dynamics simulation trajectory of the 5-methoxy tryptophan mutant of PhoSL in complex with core fucose N-glycans. The three PhoSL protein monomers are depicted using ribbons colored green, cyan, and magenta. The core fucose N-glycan residues are shown as sticks with varying colors. The 5-methoxy tryptophan residues are represented by spheres.

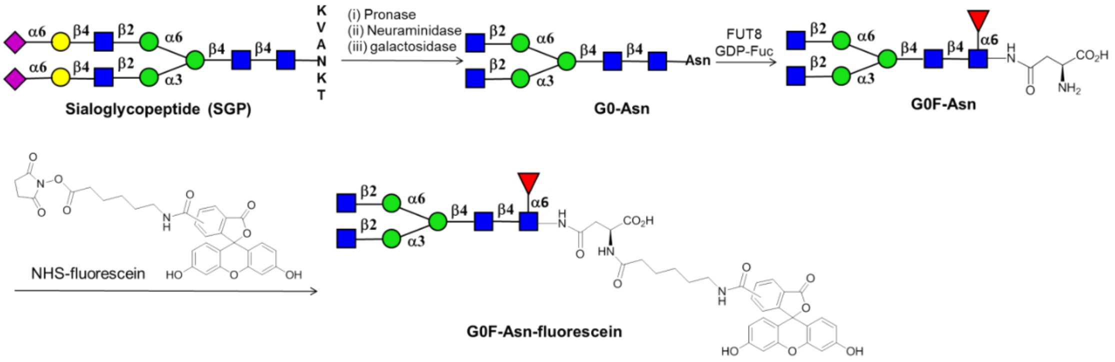

Figure S1. The preparation of G0F-Asn-fluorescein **1** (synthetic details see Methods)

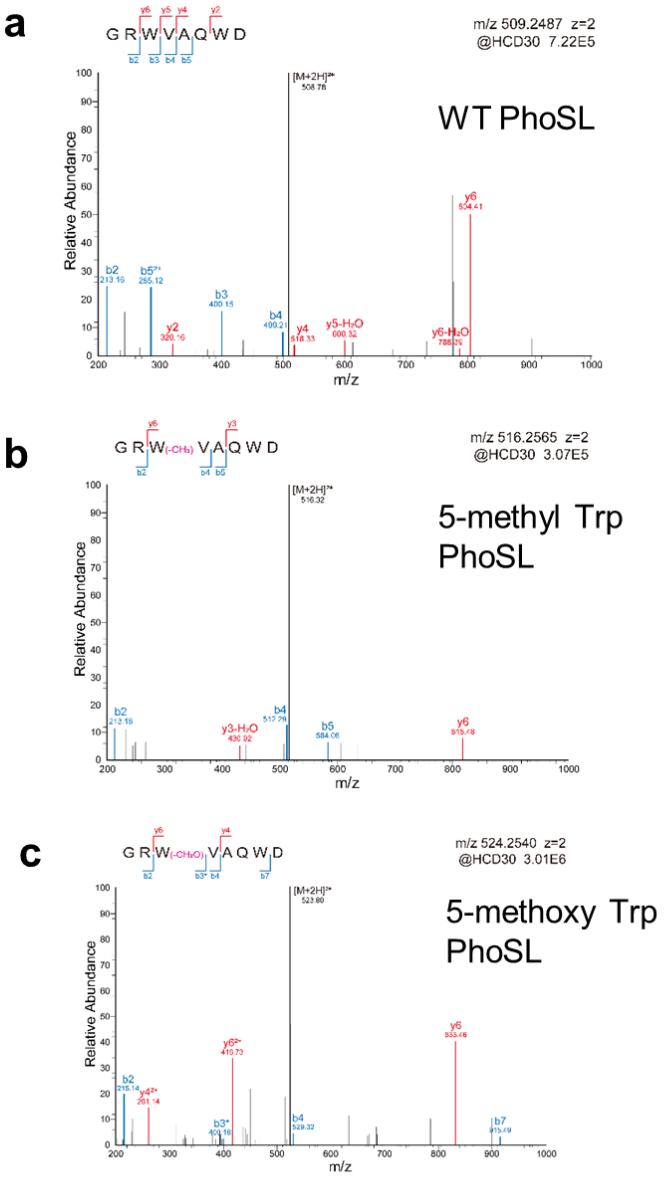

Figure S2. Site mapping of the modification sites of WT (a), 5-methyl Trp incorporated (b), and 5-methoxy Trp incorporated (c) PhoSL.

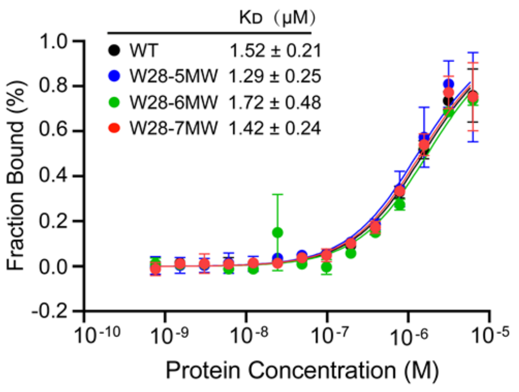

Figure S3. The binding affinity of the WT and different variants of PhoSL toward the core fucose glycan substrate **1** as measured by microscale thermophoresis. Error bars denote the means ± SD in three independent assays.

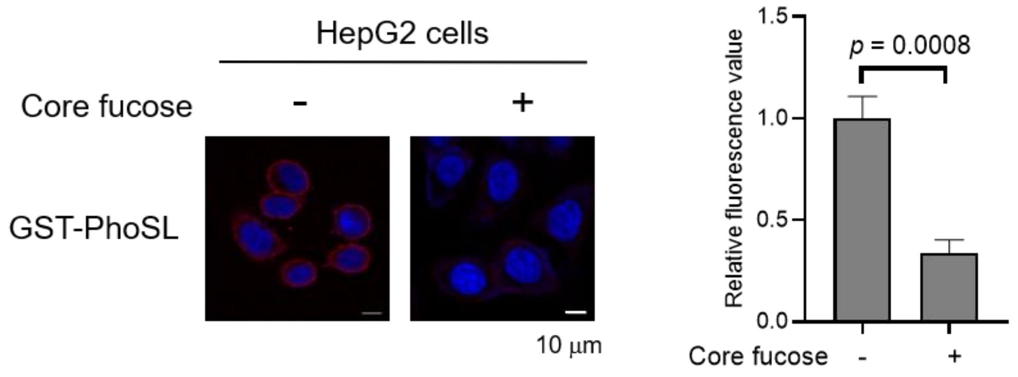

Figure S4. Immunofluorescence analysis of core fucose glycans on HepG2 cell surface using GST-tagged PhoSL in the presence or absence of the core fucose substrate. The relative values were determined by Image J (n= 3 independent assays). Scale bar: 10 µm. Error bars denote the mean ± SD. Statistical analyses were performed by unpaired two-tailed Student's t-tests.

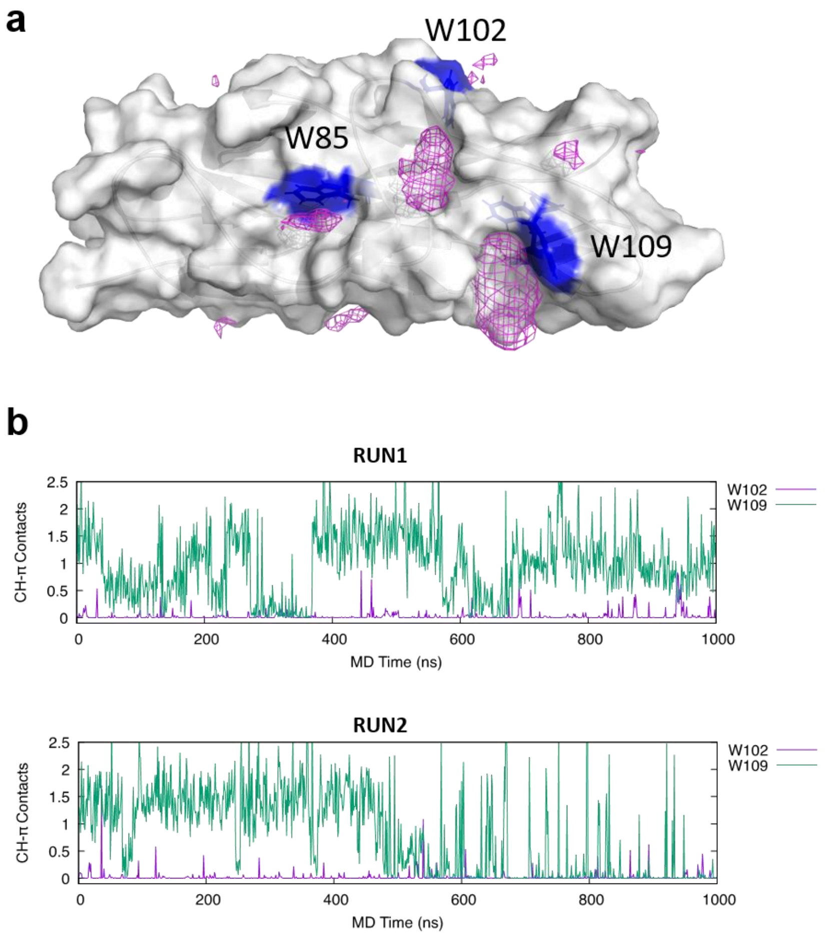

Figure S5. **W109 is a critical residue for GlcNAc-containing glycan recognition by GafD.** (a), The energy minimized crystal structure of the glycan binding site of GafD in complex with the GlcNAc-containing glycan (PDB: 1OI0). (b), The CH-π contacts between the aromatic residues W102 and W109 of GafD and the CH groups of the glycan, shown as a function of simulation time in two independent 1000 ns MD simulations.

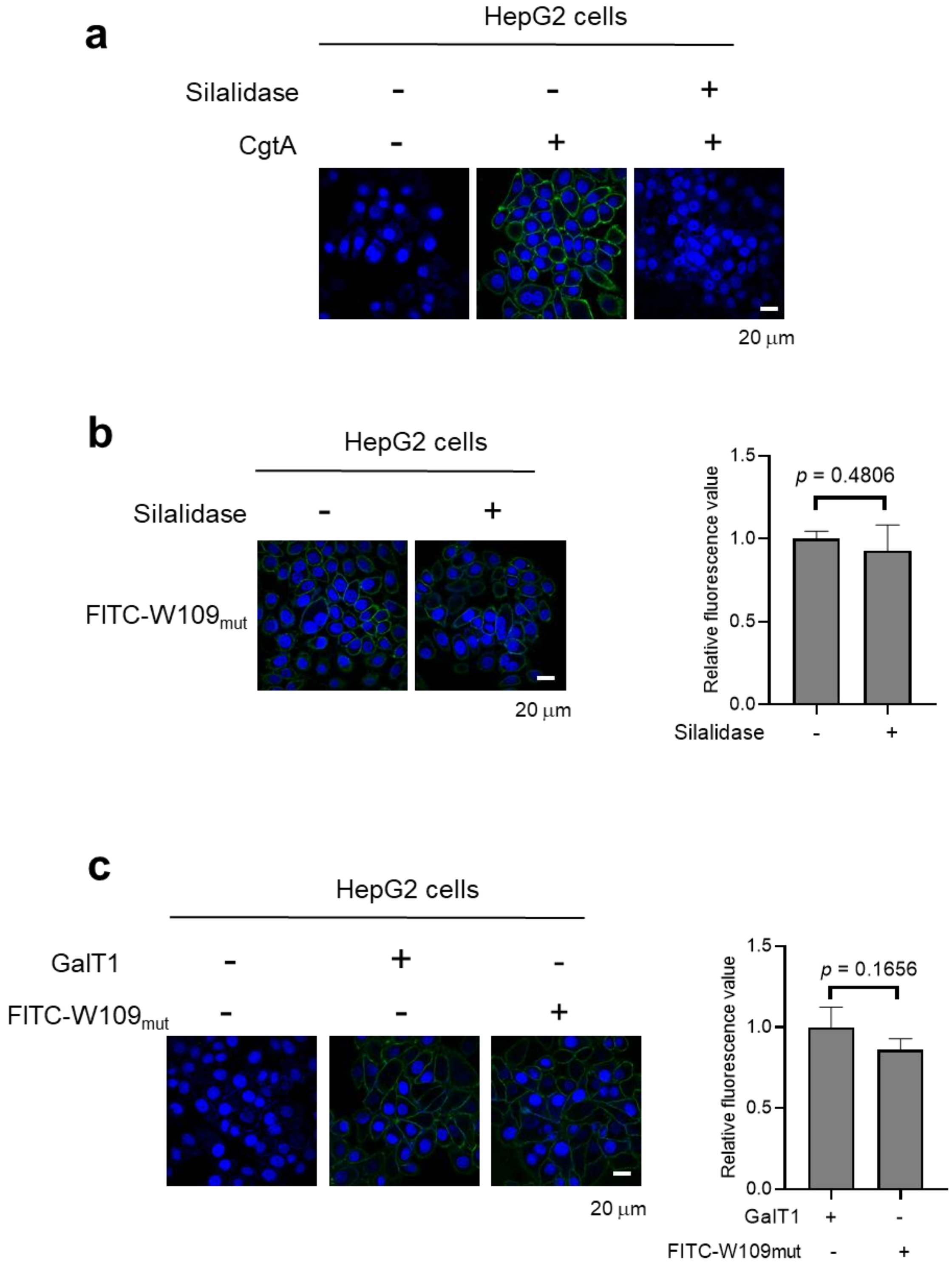

Figure S6. (a), Immunofluorescence analysis of sialylation on the cell surface by a CgtA mediated chemoenzymatic labeling strategy. (b), Immunofluorescence analysis of GlcNAc-containing glycans on HepG2 cell surface in the presence or absence of silalidase. (c), Immunofluorescence analysis of GlcNAc-containing glycans on HepG2 cell surface using

GalT1mediated chemoenzymatic labeling strategy or FITC-W109$_{mut}$. The relative values were determined by Image J (n= 3 independent assays). Scale bar: 10 µm. Error bars denote the mean ± SD. Statistical analyses were performed by unpaired two-tailed Student's t-tests.

Compound characterization spectra

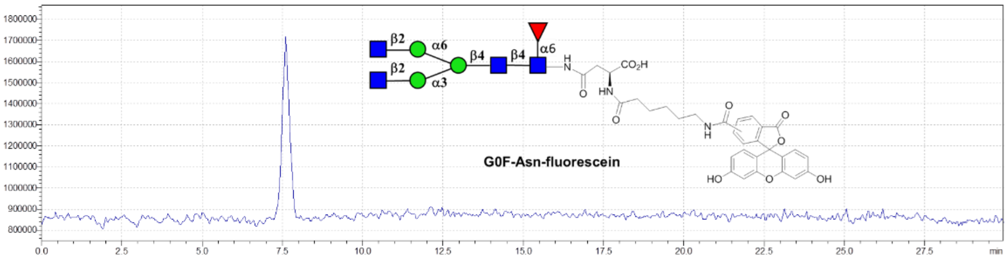

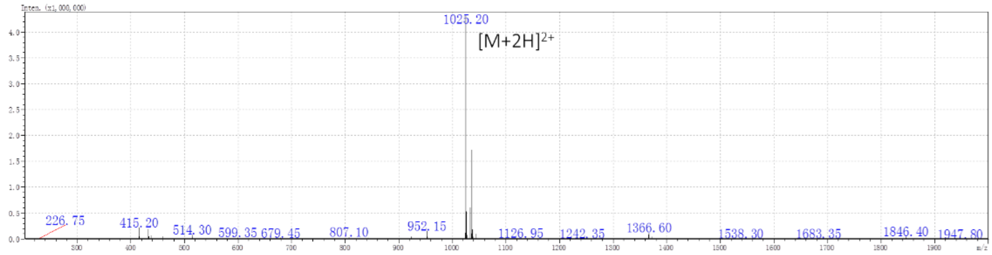

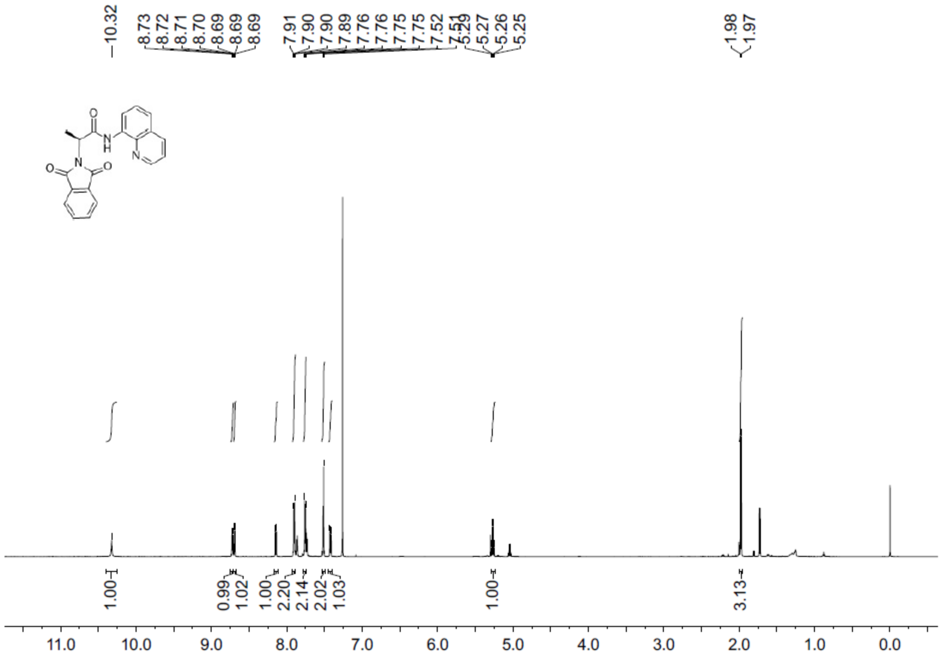

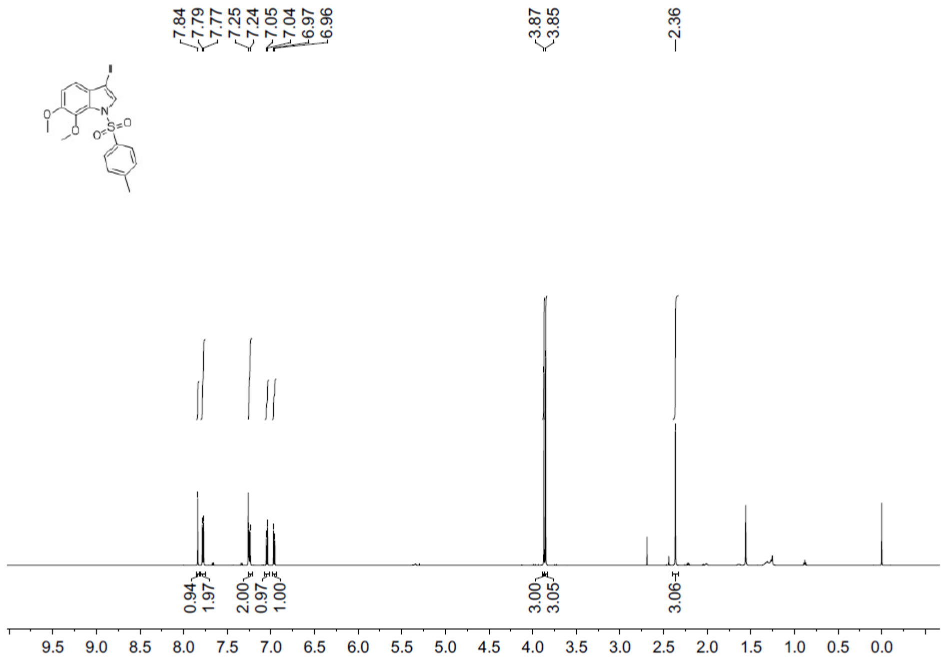
7.84
7.79
7.77
7.25
7.24
7.05
7.04
6.97
6.96
3.87
3.85
2.36
0.94
1.97
2.00
0.97
1.00
3.00
3.05
3.06
9.5 9.0 8.5 8.0 7.5 7.0 6.5 6.0 5.5 5.0 4.5 4.0 3.5 3.0 2.5 2.0 1.5 1.0 0.5 0.0

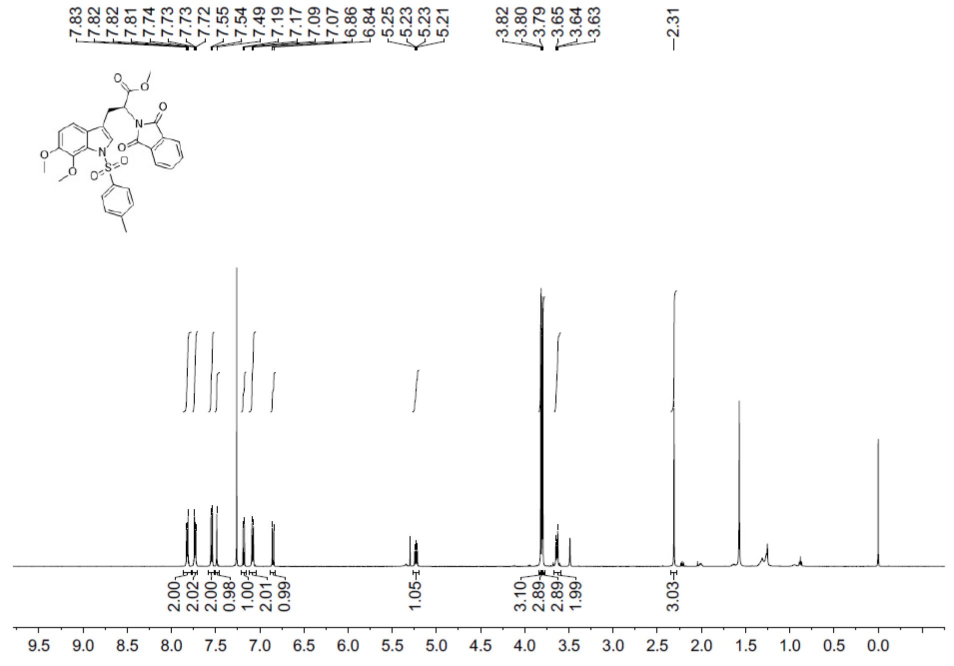
7.83
7.82
7.82
7.81
7.74
7.73
7.73
7.72
7.55
7.54
7.49
7.19
7.17
7.09
7.07
6.86
6.84
5.25
5.23
5.23
5.21
3.82
3.80
3.79
3.65
3.64
3.63
2.31
2.00
2.02
2.00
0.98
1.00
2.01
0.99
1.05
3.10
2.89
2.89
1.99
3.03
9.5 9.0 8.5 8.0 7.5 7.0 6.5 6.0 5.5 5.0 4.5 4.0 3.5 3.0 2.5 2.0 1.5 1.0 0.5 0.0

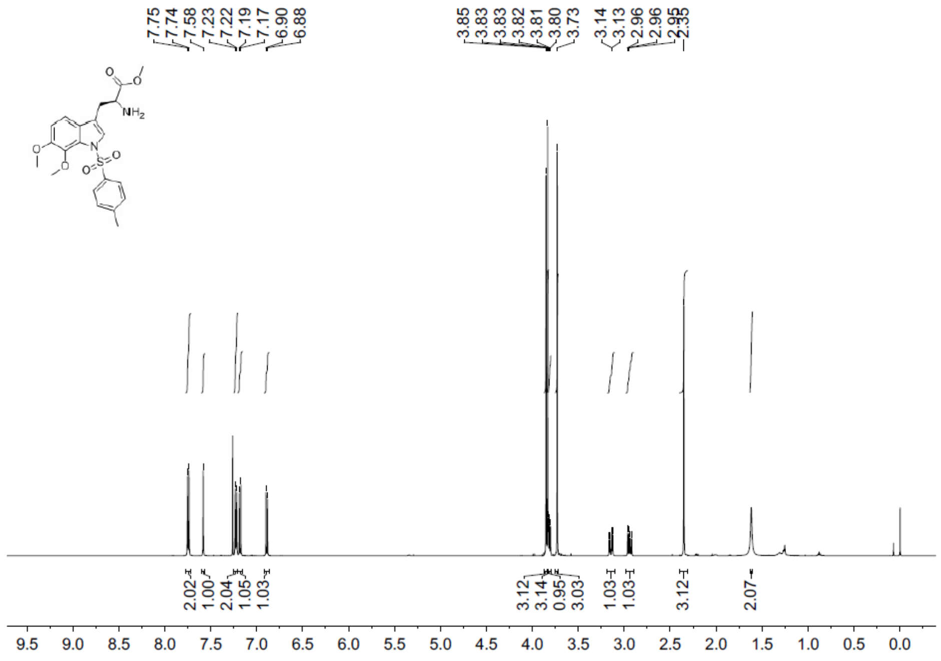

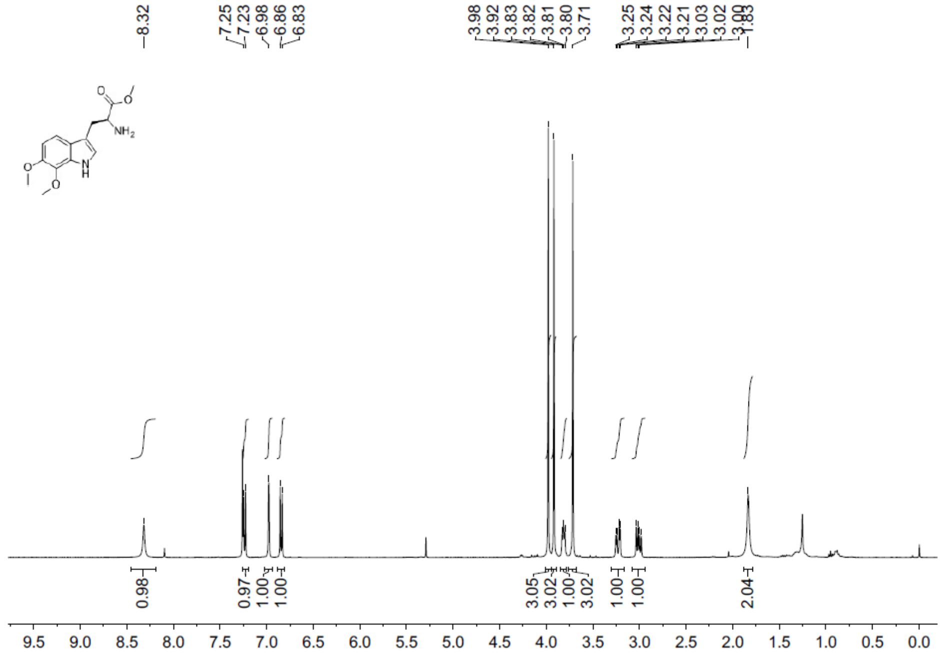

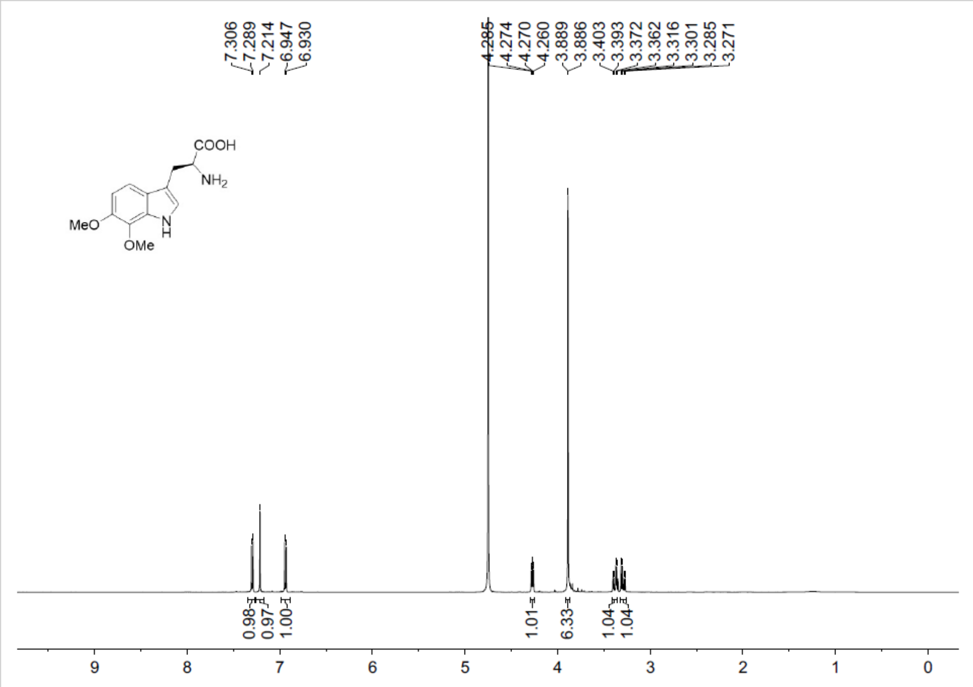

7.306
7.289
7.214
6.947
6.930
4.285
4.274
4.270
4.260
3.889
3.886
3.403
3.393
3.372
3.362
3.316
3.301
3.285
3.271
COOH
NH2
MeO
OMe
H
N
0.98
0.97
1.00
1.01
6.33
1.04
1.04
9
8
7
6
5
4
3
2
1
0

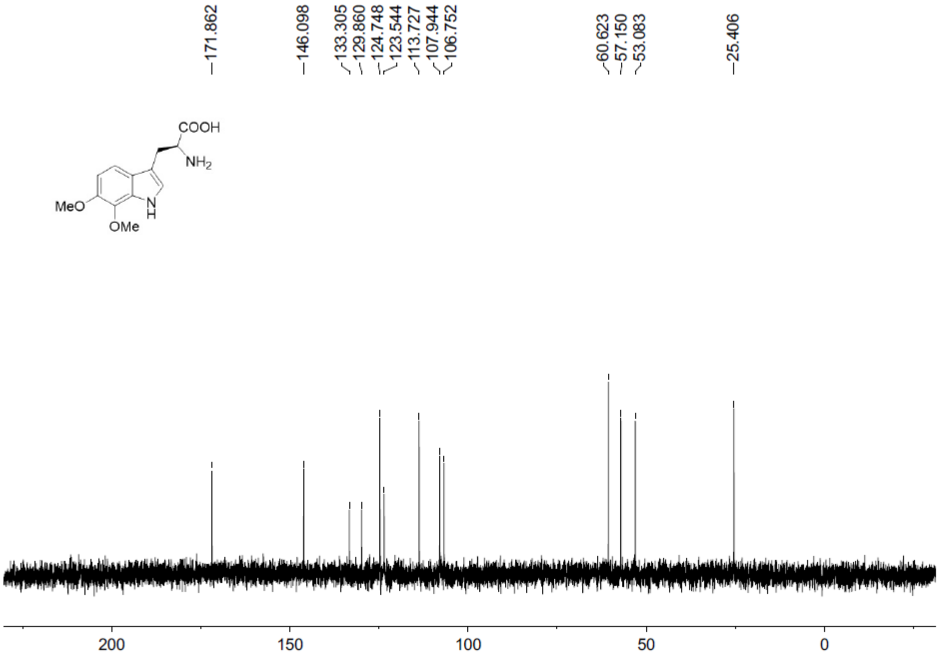

171.862
146.098
133.305
129.860
124.748
123.544
113.727
107.944
106.752
60.623
57.150
53.083
25.406
COOH
NH2
MeO
OMe
H
N
200
150
100
50
0

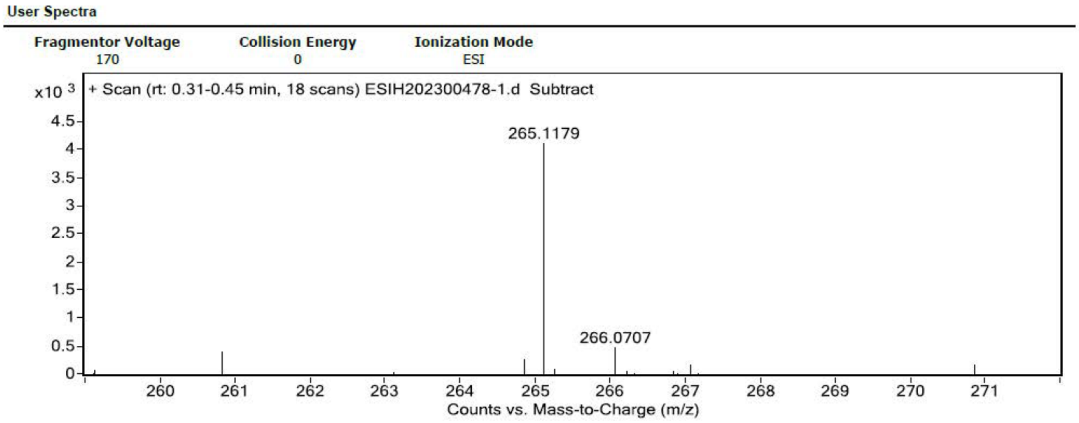

Formula Calculator Results

m/z	Calc m/z	Diff (mDa)	Diff (ppm)	Ion Formula	Ion
265.1179	265.1183	0.41	1.53	C13 H17 N2 O4	(M+H)+

--- End Of Report ---

Table S1. 451 core-fucosylated proteins were identified in HepG2 cells.

Uniprot ID	Gene names	Overlap with Cao *et al.* (Nature Communications, 2022)	Overlap with Jia *et al.* (Theranostics, 2021)	Overlap with Luo *et al.* (Journal of the American Chemical Society, 2023)
Q8NBJ5	COLGALT1	YES	YES	YES
Q4KMQ2	ANO6	YES	YES	YES
Q10589	BST2	YES	YES	NO
P08195	SLC3A2	YES	YES	YES
P07339	CTSD	YES	YES	NO
Q08722	CD47	YES	YES	YES
Q9Y639	NPTN	YES	YES	YES
Q6UVK1	CSPG4	YES	YES	NO
P00533	EGFR	YES	YES	YES
P35613	BSG	YES	YES	YES
Q14126	DSG2	YES	YES	YES
P78406	RAE1	YES	YES	NO
P32004	L1CAM	YES	YES	YES
P11717	IGF2R	YES	YES	YES
P15529	CD46	YES	YES	NO
P26006	ITGA3	YES	YES	YES
P16070	CD44	YES	YES	NO
P06756	ITGAV	YES	YES	YES
P05556	ITGB1	YES	YES	YES
P48960	CD97	YES	YES	NO
P56199	ITGA1	YES	YES	YES
Q14571	ITPR2	YES	YES	YES
Q6YHK3	CD109	YES	YES	YES
Q13740	ALCAM	YES	YES	YES
Q9P2B2	PTGFRN	YES	YES	NO
P43121	MCAM	YES	YES	NO
Q6P4Q7	CNNM4	YES	YES	NO
Q07954	LRP1	YES	YES	YES

P08648	ITGA5	YES	YES	YES
P17301	ITGA2	YES	YES	YES
P06213	INSR	YES	YES	YES
O94901	SUN1	YES	YES	YES
P20645	M6PR	YES	YES	YES
P19022	CDH2	YES	YES	YES
Q9UGT4	SUSD2	YES	YES	NO
Q9Y6N7	ROBO1	YES	YES	YES
P18084	ITGB5	YES	YES	YES
Q92508	PIEZO1	YES	YES	NO
P53634	CTSC	YES	YES	YES
O00469	PLOD2	YES	YES	YES
P05362	ICAM1	YES	YES	YES
P23229	ITGA6	YES	YES	YES
Q9Y4D7	PLXND1	YES	YES	NO
O15031	PLXNB2	YES	YES	YES
Q9BVK6	TMED9	YES	YES	YES
Q15181	PPA1	YES	NO	NO
Q13263	TRIM28	YES	NO	NO
Q7Z6Z7	HUWE1	YES	NO	NO
O60763	USO1	YES	NO	NO
Q14980	NUMA1	YES	NO	NO
O94906	PRPF6	YES	NO	NO
Q9NZM1	MYOF	YES	NO	NO
Q14118	DAG1	YES	NO	YES
Q99614	TTC1	YES	NO	NO
Q13501	SQSTM1	YES	NO	NO
Q9NQC3	RTN4	YES	NO	NO
P29144	TPP2	YES	NO	NO
Q12769	NUP160	YES	NO	NO
Q8TAQ2	SMARCC2	YES	NO	NO
Q92945	KHSRP	YES	NO	NO
Q7Z2K6	ERMP1	YES	NO	NO
Q13813	SPTAN1	YES	NO	NO
P52948	NUP98	YES	NO	NO
P17655	CAPN2	YES	NO	NO
P04632	CAPNS1	YES	NO	NO
Q6P2Q9	PRPF8	YES	NO	NO
Q96I24	FUBP3	YES	NO	NO
P51610	HCFC1	YES	NO	NO
Q63HN8	RNF213	YES	NO	NO
Q14008	CKAP5	YES	NO	NO
P08581	MET	YES	NO	1
Q8NBU5	ATAD1	YES	NO	NO
Q96ST3	SIN3A	YES	NO	NO
Q6P2E9	EDC4	YES	NO	NO
P33176	KIF5B	YES	NO	NO
Q9UPN3	MACF1	YES	NO	NO
Q8NC42	RNF149	YES	NO	YES
Q14157	UBAP2L	YES	NO	NO
Q5VT52	RPRD2	YES	NO	NO

P46939	UTRN	YES	NO	NO
P42285	SKIV2L2	YES	NO	NO
Q14789	GOLGB1	YES	NO	NO
Q6P1M0	SLC27A4	YES	NO	NO
P42765	ACAA2	YES	NO	NO
Q9H0X4	ITFG3	YES	NO	YES
P56537	EIF6	YES	NO	NO
Q13561	DCTN2	YES	NO	NO
P17813	ENG	YES	NO	NO
P52294	KPNA1	YES	NO	NO
Q99808	SLC29A1	YES	NO	NO
Q5JRA6	MIA3	YES	NO	YES
Q12913	PTPRJ	YES	NO	YES
P61160	ACTR2	YES	NO	NO
Q9UHD8	SEPT9	YES	NO	NO
Q15369	TCEB1	YES	NO	NO
O75179	ANKRD17	YES	NO	NO
Q9UPN9	TRIM33	YES	NO	NO
Q13620	CUL4B	YES	NO	NO
P38435	GGCX	YES	NO	YES
P51116	FXR2	YES	NO	NO
Q9NTJ5	SACM1L	YES	NO	NO
Q687X5	STEAP4	YES	NO	NO
Q7L2H7	EIF3M	YES	NO	NO
O75694	NUP155	YES	NO	NO
Q9Y4P3	TBL2	YES	NO	NO
O60716	CTNND1	YES	NO	NO
P51649	ALDH5A1	YES	NO	NO
Q8WTV0	SCARB1	YES	NO	YES
Q9Y223	GNE	YES	NO	NO
Q96RQ1	ERGIC2	YES	NO	NO
Q9ULC5	ACSL5	YES	NO	NO
Q9NX40	OCIAD1	YES	NO	NO
O94855	SEC24D	YES	NO	NO
O75907	DGAT1	YES	NO	NO
Q53H82	LACTB2	YES	NO	NO
Q93100	PHKB	YES	NO	NO
P30530	AXL	YES	NO	NO
Q9H4A4	RNPEP	YES	NO	NO
Q709C8	VPS13C	NO	YES	NO
P05187	ALPP	NO	YES	NO
P61011	SRP54	NO	YES	NO
P46379	BAG6	NO	YES	NO
O75534	CSDE1	NO	YES	NO
P08174	CD55	NO	YES	NO
O00592	PODXL	NO	YES	NO
P15151	PVR	NO	YES	YES
P48509	CD151	NO	YES	NO
P33527	ABCC1	NO	YES	NO
P12270	TPR	NO	YES	NO
Q9BWF3	RBM4	NO	YES	NO

Q5JPE7	NOMO2	NO	YES	NO
O75592	MYCBP2	NO	YES	NO
Q7Z460	CLASP1	NO	YES	NO
Q7Z3B4	NUP54	NO	YES	NO
Q92769	HDAC2	NO	NO	NO
P46778	RPL21	NO	NO	NO
Q13286	CLN3	NO	NO	NO
Q6DD88	ATL3	NO	NO	NO
Q01650	SLC7A5	NO	NO	NO
P49755	TMED10	NO	NO	NO
P10398	ARAF	NO	NO	NO
P48775	TDO2	NO	NO	NO
Q8NFJ5	GPRC5A	NO	NO	NO
Q92643	PIGK	NO	NO	NO
Q14318	FKBP8	NO	NO	NO
Q8WUY1	THEM6	NO	NO	NO
Q5SRE5	NUP188	NO	NO	NO
Q6PGP7	TTC37	NO	NO	NO
P37235	HPCAL1	NO	NO	NO
Q9Y6C9	MTCH2	NO	NO	NO
P30043	BLVRB	NO	NO	NO
P42345	MTOR	NO	NO	NO
Q9H4L4	SENP3	NO	NO	NO
O75531	BANF1	NO	NO	NO
O00232	PSMD12	NO	NO	NO
Q9UKN8	GTF3C4	NO	NO	NO
Q9H3U1	UNC45A	NO	NO	NO
P46013	MKI67	NO	NO	NO
Q9BW27	NUP85	NO	NO	NO
Q16850	CYP51A1	NO	NO	NO
P43007	SLC1A4	NO	NO	NO
Q9Y3L5	RAP2C	NO	NO	NO
P36957	DLST	NO	NO	NO
Q9BTX1	NDC1	NO	NO	NO
Q01628	IFITM3	NO	NO	NO
Q14738	PPP2R5D	NO	NO	NO
Q9Y6Y8	SEC23IP	NO	NO	NO
Q5VYK3	ECM29	NO	NO	NO
Q9Y224	C14orf166	NO	NO	NO
Q15021	NCAPD2	NO	NO	NO
P58107	EPPK1	NO	NO	NO
Q14697	GANAB	NO	NO	NO
O96008	TOMM40	NO	NO	NO
O43169	CYB5B	NO	NO	NO
Q29RF7	PDS5A	NO	NO	NO
O75165	DNAJC13	NO	NO	NO
Q9NX47	MARCHF5	NO	NO	NO
Q9UK22	FBXO2	NO	NO	NO
Q969Z0	TBRG4	NO	NO	NO
P23634	ATP2B4	NO	NO	NO
A5YKK6	CNOT1	NO	NO	NO

Q9HAV4	XPO5	NO	NO	NO
P51812	RPS6KA3	NO	NO	NO
Q9UJX3	ANAPC7	NO	NO	NO
Q6NUQ4	TMEM214	NO	NO	NO
P53992	SEC24C	NO	NO	NO
O00410	IPO5	NO	NO	NO
O43143	DHX15	NO	NO	NO
P53396	ACLY	NO	NO	NO
Q92621	NUP205	NO	NO	NO
Q8NBM4	UBAC2	NO	NO	NO
P53794	SLC5A3	NO	NO	NO
O75648	TRMU	NO	NO	NO
Q96SK2	TMEM209	NO	NO	NO
Q6NUT3	MFSD12	NO	NO	NO
Q99567	NUP88	NO	NO	NO
Q8TCT9	HM13	NO	NO	NO
Q9UGP8	SEC63	NO	NO	NO
P84085	ARF5	NO	NO	NO
Q86W42	THOC6	NO	NO	NO
P15941	MUC1	NO	NO	NO
P55265	ADAR	NO	NO	NO
Q92600	RQCD1	NO	NO	NO
Q9UI26	IPO11	NO	NO	NO
Q9Y6M7	SLC4A7	NO	NO	YES
P62330	ARF6	NO	NO	NO
P37268	FDFT1	NO	NO	NO
O43390	HNRNPR	NO	NO	NO
Q08752	PPID	NO	NO	NO
Q9BZZ5	API5	NO	NO	NO
Q13325	IFIT5	NO	NO	NO
Q09161	NCBP1	NO	NO	NO
Q9Y262	EIF3L	NO	NO	NO
Q04760	GLO1	NO	NO	NO
Q92538	GBF1	NO	NO	NO
P22234	PAICS	NO	NO	NO
Q5SY16	NOL9	NO	NO	NO
Q9UBQ5	EIF3K	NO	NO	NO
Q9NVI1	FANCI	NO	NO	NO
O43684	BUB3	NO	NO	NO
O43688	PPAP2C	NO	NO	NO
P30260	CDC27	NO	NO	NO
Q9H4A3	WNK1	NO	NO	NO
P60033	CD81	NO	NO	NO
O14880	MGST3	NO	NO	NO
O14929	HAT1	NO	NO	NO
Q9NPQ8	RIC8A	NO	NO	NO
Q92734	TFG	NO	NO	NO
Q7L5D6	GET4	NO	NO	NO
Q13547	HDAC1	NO	NO	NO
Q14534	SQLE	NO	NO	NO
Q99615	DNAJC7	NO	NO	NO

Q02218	OGDH	NO	NO	NO
Q9BTV4	TMEM43	NO	NO	NO
P57678	GEMIN4	NO	NO	NO
Q9H2H9	SLC38A1	NO	NO	NO
O00560	SDCBP	NO	NO	NO
Q9BUP3	HTATIP2	NO	NO	NO
Q15459	SF3A1	NO	NO	NO
Q6R327	RICTOR	NO	NO	NO
O95139	NDUFB6	NO	NO	NO
Q14573	ITPR3	NO	NO	NO
P61289	PSME3	NO	NO	NO
P55735	SEC13	NO	NO	NO
P37198	NUP62	NO	NO	NO
Q9BYX2	TBC1D2	NO	NO	NO
O75367	H2AFY	NO	NO	NO
P49821	NDUFV1	NO	NO	NO
O75643	SNRNP200	NO	NO	NO
O15397	IPO8	NO	NO	NO
P41223	BUD31	NO	NO	NO
Q5UIP0	RIF1	NO	NO	NO
Q15393	SF3B3	NO	NO	NO
P57740	NUP107	NO	NO	NO
Q96KR1	ZFR	NO	NO	NO
P30519	HMOX2	NO	NO	NO
O95714	HERC2	NO	NO	NO
Q9Y6M5	SLC30A1	NO	NO	NO
Q9UNQ0	ABCG2	NO	NO	NO
P43304	GPD2	NO	NO	NO
Q9Y4W2	LAS1L	NO	NO	NO
Q07864	POLE	NO	NO	NO
P16152	CBR1	NO	NO	NO
Q8IX12	CCAR1	NO	NO	NO
O95239	KIF4A	NO	NO	NO
P61769	B2M	NO	NO	NO
Q9ULX6	AKAP8L	NO	NO	NO
Q9BVL2	NUPL1	NO	NO	NO
Q9Y6D5	ARFGEF2	NO	NO	NO
P56282	POLE2	NO	NO	NO
Q9BXB5	OSBPL10	NO	NO	NO
O60664	PLIN3	NO	NO	NO
Q9UPY5	SLC7A11	NO	NO	NO
P04920	SLC4A2	NO	NO	NO
Q9H583	HEATR1	NO	NO	NO
Q9UNE7	STUB1	NO	NO	NO
Q99873	PRMT1	NO	NO	NO
Q9H1K1	ISCU	NO	NO	NO
O95453	PARN	NO	NO	YES
O43670	ZNF207	NO	NO	NO
Q9BQA1	WDR77	NO	NO	NO
Q08379	GOLGA2	NO	NO	NO
Q9Y289	SLC5A6	NO	NO	NO

Q14139	UBE4A	NO	NO	NO
O95336	PGLS	NO	NO	NO
P00492	HPRT1	NO	NO	NO
Q9UGI8	TES	NO	NO	NO
Q9H3S7	PTPN23	NO	NO	NO
O60566	BUB1B	NO	NO	NO
P57105	SYNJ2BP	NO	NO	NO
Q8ND04	SMG8	NO	NO	NO
O75683	SURF6	NO	NO	NO
Q9BZK7	TBL1XR1	NO	NO	NO
O14744	PRMT5	NO	NO	NO
Q7Z4H7	HAUS6	NO	NO	NO
Q9UDY8	MALT1	NO	NO	NO
P46087	NOP2	NO	NO	NO
Q15058	KIF14	NO	NO	NO
Q13315	ATM	NO	NO	NO
Q9BXW9	FANCD2	NO	NO	NO
O15403	SLC16A6	NO	NO	NO
P69905	HBA1	NO	NO	NO
O60645	EXOC3	NO	NO	NO
Q9BTE3	MCMBP	NO	NO	NO
Q86VM9	ZC3H18	NO	NO	NO
Q9UJX4	ANAPC5	NO	NO	NO
Q9HD33	MRPL47	NO	NO	NO
Q9ULQ1	TPCN1	NO	NO	NO
Q15022	SUZ12	NO	NO	NO
Q14690	PDCD11	NO	NO	NO
Q9UNN5	FAF1	NO	NO	NO
Q32MZ4	LRRFIP1	NO	NO	NO
O94822	LTN1	NO	NO	NO
Q7L1W4	LRRC8D	NO	NO	NO
O15372	EIF3H	NO	NO	NO
P19784	CSNK2A2	NO	NO	NO
Q12788	TBL3	NO	NO	NO
O14646	CHD1	NO	NO	NO
P51532	SMARCA4	NO	NO	NO
Q5SNT2	TMEM201	NO	NO	NO
Q8IWT6	LRRC8A	NO	NO	NO
O95169	NDUFB8	NO	NO	NO
Q8IZV5	RDH10	NO	NO	NO
P16066	NPR1	NO	NO	NO
Q15751	HERC1	NO	NO	NO
Q6NXE6	ARMC6	NO	NO	NO
Q9C0D5	TANC1	NO	NO	NO
Q9UHY1	NRBP1	NO	NO	NO
Q9UPT5	EXOC7	NO	NO	NO
A6NHR9	SMCHD1	NO	NO	NO
Q13409	DYNC1I2	NO	NO	NO
P61201	COPS2	NO	NO	NO
P62995	TRA2B	NO	NO	NO
Q9UKX7	NUP50	NO	NO	NO

Q96C36	PYCR2	NO	NO	NO
Q9Y3E5	PTRH2	NO	NO	NO
Q9BVG8	KIFC3	NO	NO	NO
P30876	POLR2B	NO	NO	NO
Q8IUC1	KRTAP11-1	NO	NO	NO
Q8NEB9	PIK3C3	NO	NO	NO
A6NDG6	PGP	NO	NO	NO
Q16181	SEPT7	NO	NO	NO
P24928	POLR2A	NO	NO	NO
O60518	RANBP6	NO	NO	NO
O60318	MCM3AP	NO	NO	NO
Q96EE3	SEH1L	NO	NO	NO
Q9BY44	EIF2A	NO	NO	NO
P31641	SLC6A6	NO	NO	NO
Q9UL15	BAG5	NO	NO	NO
P55036	PSMD4	NO	NO	NO
P55017	SLC12A3	NO	NO	NO
Q5H9R7	PPP6R3	NO	NO	NO
O60825	PFKFB2	NO	NO	NO
Q8N2K0	ABHD12	NO	NO	NO
Q8IWZ3	ANKHD1	NO	NO	NO
P46934	NEDD4	NO	NO	NO
P40222	TXLNA	NO	NO	NO
O60503	ADCY9	NO	NO	NO
O00541	PES1	NO	NO	NO
Q9BT78	COPS4	NO	NO	NO
Q9BW92	TARS2	NO	NO	NO
Q53EL6	PDCD4	NO	NO	NO
Q8NI60	ADCK3	NO	NO	NO
Q9BVS4	RIOK2	NO	NO	NO
O15438	ABCC3	NO	NO	NO
Q8N3C0	ASCC3	NO	NO	NO
Q9Y5Y0	FLVCR1	NO	NO	NO
Q6KC79	NIPBL	NO	NO	NO
P33897	ABCD1	NO	NO	NO
Q9BUB7	TMEM70	NO	NO	NO
P61421	ATP6V0D1	NO	NO	NO
Q12800	TFCP2	NO	NO	NO
P82933	MRPS9	NO	NO	NO
P10644	PRKAR1A	NO	NO	NO
Q53H12	AGK	NO	NO	NO
Q96BD0	SLCO4A1	NO	NO	NO
Q9H8Y8	GORASP2	NO	NO	NO
A8MPP1	DDX11L8	NO	NO	NO
Q9Y6E0	STK24	NO	NO	NO
Q9HC21	SLC25A19	NO	NO	NO
Q8TCG1	KIAA1524	NO	NO	NO
Q86XL3	ANKLE2	NO	NO	NO
Q93050	ATP6V0A1	NO	NO	NO
P55039	DRG2	NO	NO	NO
Q13395	TARBP1	NO	NO	NO

P41440	SLC19A1	NO	NO	NO
P42025	ACTR1B	NO	NO	NO
Q96NT5	SLC46A1	NO	NO	NO
P48147	PREP	NO	NO	NO
Q03519	TAP2	NO	NO	NO
Q13188	STK3	NO	NO	NO
Q14914	PTGR1	NO	NO	NO
P20839	IMPDH1	NO	NO	NO
Q15599	SLC9A3R2	NO	NO	NO
Q9BXW7	CECR5	NO	NO	NO
Q9H0H5	RACGAP1	NO	NO	NO
Q9GZZ1	NAA50	NO	NO	NO
P17480	UBTF	NO	NO	NO
Q53EP0	FNDC3B	NO	NO	NO
Q9P015	MRPL15	NO	NO	NO
Q15286	RAB35	NO	NO	NO
Q6IAN0	DHRS7B	NO	NO	NO
Q96A49	SYAP1	NO	NO	NO
Q9UII4	HERC5	NO	NO	NO
P48449	LSS	NO	NO	NO
O14734	ACOT8	NO	NO	NO
Q14692	BMS1	NO	NO	NO
P30085	CMPK1	NO	NO	NO
Q9BZE1	MRPL37	NO	NO	NO
Q9HCU5	PREB	NO	NO	NO
P83111	LACTB	NO	NO	NO
Q7L8L6	FASTKD5	NO	NO	NO
P20585	MSH3	NO	NO	NO
Q9NP79	VTA1	NO	NO	NO
P18754	RCC1	NO	NO	NO
Q8IWA4	MFN1	NO	NO	NO
Q7Z2T5	TRMT1L	NO	NO	NO
Q15785	TOMM34	NO	NO	NO
O14964	HGS	NO	NO	NO
Q13769	THOC5	NO	NO	NO
O00411	POLRMT	NO	NO	NO
O95071	UBR5	NO	NO	NO
Q96SB4	SRPK1	NO	NO	NO
O95155	UBE4B	NO	NO	NO
P35237	SERPINB6	NO	NO	NO
O60443	DFNA5	NO	NO	NO
Q15005	SPCS2	NO	NO	NO
Q15126	PMVK	NO	NO	NO
P42695	NCAPD3	NO	NO	NO
Q08AM6	VAC14	NO	NO	NO
Q01433	AMPD2	NO	NO	NO
O15228	GNPAT	NO	NO	NO
Q86X55	CARM1	NO	NO	NO
Q9NXF1	TEX10	NO	NO	NO
Q96S55	WRNIP1	NO	NO	NO
Q8IWX8	CHERP	NO	NO	NO

Q13347	EIF3I	NO	NO	NO
P50995	ANXA11	NO	NO	NO
Q96R06	SPAG5	NO	NO	NO
Q9Y6G9	DYNC1LI1	NO	NO	NO
Q92520	FAM3C	NO	NO	NO
Q1KMD3	HNRNPUL2	NO	NO	NO
P18433	PTPRA	NO	NO	NO
P43003	SLC1A3	NO	NO	NO
Q15800	MSMO1	NO	NO	NO
O95870	ABHD16A	NO	NO	NO
P48067	SLC6A9	NO	NO	NO
O15431	SLC31A1	NO	NO	NO
Q92879	CELF1	NO	NO	NO
O15294	OGT	NO	NO	NO
P11388	TOP2A	NO	NO	NO
